# Construction of TLS-related gene signature for predicting prognosis and immunotherapy response in hepatocellular carcinoma

**DOI:** 10.1097/HC9.0000000000000925

**Published:** 2026-03-26

**Authors:** Jian-Rong Li, Li-Xin Pan, Jia-Yong Su, Wei Tian, Lei-Po Lin, Zhi-Hao Huang, Cheng-Piao Luo, Zhen-Zhen Li, Liang Ma, Ping-Ping Guo, Yi-Li Ma, Jian-Hong Zhong

**Affiliations:** 1 Hepatobiliary Surgery Department, Guangxi Medical University Cancer Hospital, Nanning, China; 2 Department of Ultrasound, The FifthAffiliated Hospital, Southern Medical University, Guangzhou, China.; 3 Pathology Department, Guangxi Medical University Cancer Hospital, Nanning, China; 4 Ultrasound Department, Guangxi Medical University Cancer Hospital, Nanning, China; 5 Key Laboratory of Early Prevention and Treatment for Regional High-Frequency Tumors, Guangxi Medical University, Ministry of Education, Nanning, China; 6 Guangxi Key Laboratory of Early Prevention and Treatment for Regional High-Frequency Tumors, Nanning, China

**Keywords:** HCC, immunotherapy, prognostic signature, tertiary lymphoid structures, tumor microenvironment

## Abstract

**Background:**

Tertiary lymphoid structures (TLSs) are critical components of the tumor microenvironment in HCC. However, the role of TLS-related molecules in predicting HCC outcomes and guiding immunotherapy remains unexplored. This study aimed to develop TLS-related gene signature (TLSRS) to predict prognosis and immunotherapy response in patients with HCC​​​​​​.

**Methods:**

TLS-related genes (TLSRGs) were identified through differential gene expression analysis combined with weighted correlation network analysis. TLSRGs were selected to take the intersection and LASSO regression to construct a TLSRS. Following validation of the associations between clinical prognosis and TLS-related gene features, analyses were further expanded to GSEA, HCC molecular classes, tumor microenvironment characteristics, immune-related molecular features and immunotherapy responsiveness. TLSRS was further explored using single-cell, spatial transcriptomics, immunohistochemistry, and multiplex immunofluorescence to determine their relationship with TLS.

**Results:**

TLSs are identified as a favorable prognostic factor in HCC. Three TLSRGs (ACKR1, CCR7, and IL7R), which were associated with both TLS presence and prognosis, were constructed with TLSRS. The TLSRS-high group, which was associated with inflammation-related HCC molecular subtypes, exhibited significantly improved clinical outcomes, along with enhanced immune cell infiltration, enriched immune response pathways, and a higher probability of response to immunotherapy. Results from single-cell, spatial transcriptomics, immunohistochemistry, and multiplex immunofluorescence revealed that ACKR1, CCR7, and IL7R shared similar expression patterns and spatial localization with TLS.

**Conclusions:**

The TLSRS shows a correlation with both prognosis and immunotherapy response in patients with HCC, suggesting its potential value in advancing precision medicine for HCC.

## Graphical abstract

**Figure:**
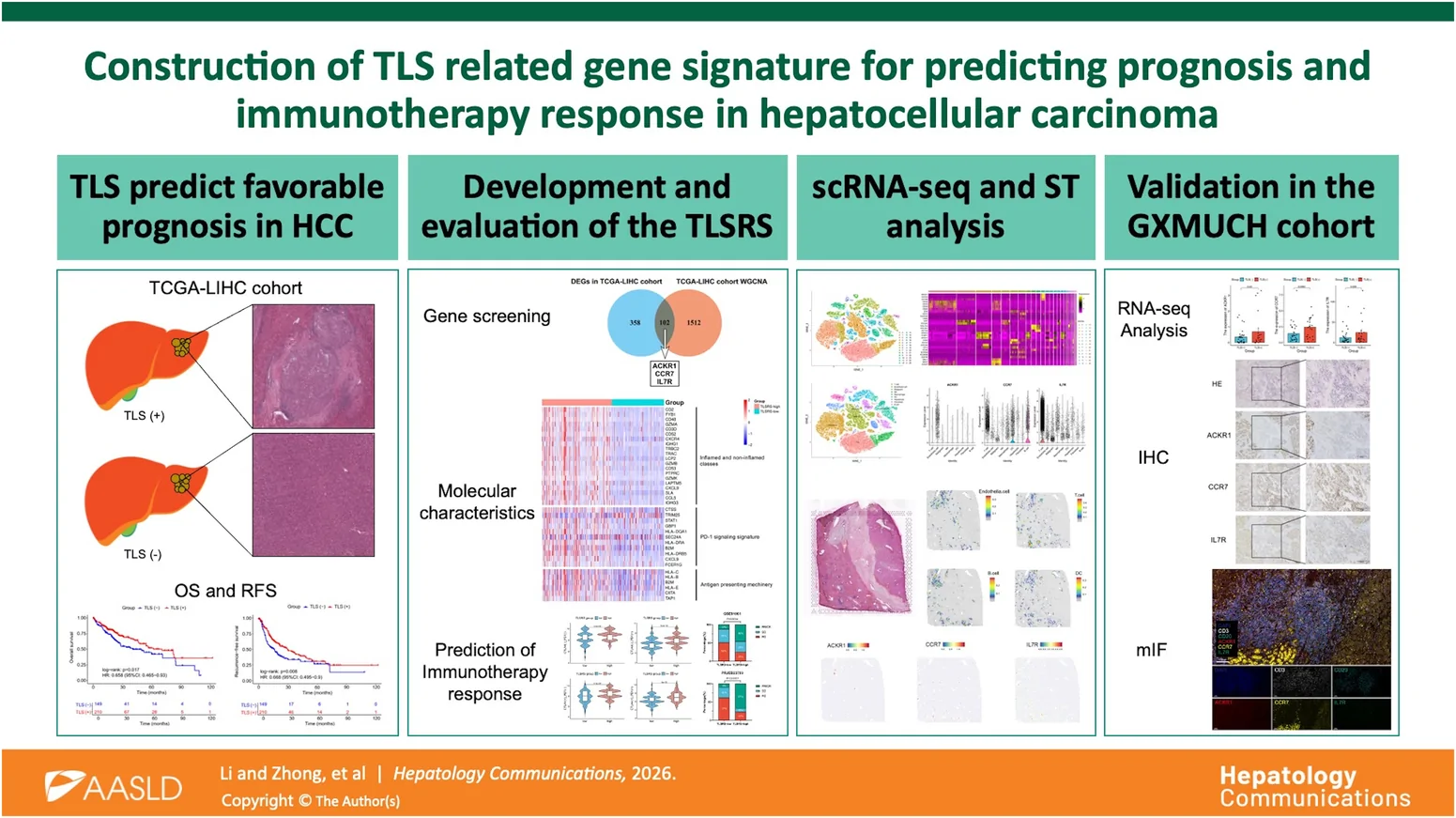

## INTRODUCTION

HCC is insidious, with most patients diagnosed at an advanced stage and only a small proportion eligible for surgical resection.^1^^,^^2^ Immune checkpoint inhibitors have recently become the standard treatment for advanced HCC. However, due to tumor heterogeneity and diverse tumor immune microenvironments, immunotherapy response rates remain unsatisfactory, with many patients still having poor prognosis.^3^^,^^4^ Thus, there is an urgent need to identify new molecular targets to boost HCC immunotherapy, enable personalized assessment of treatment response and prognosis, and ultimately improve long-term survival.

Tertiary lymphoid structures (TLSs) are lymphoid-like formations in nonlymphoid tissues, mainly in inflamed areas, linked to pathologies like chronic inflammation, autoimmunity, and cancer.^5^ In the tumor immune microenvironment, TLSs are formed by the aggregation of immune cells under the influence of various cytokines. Conversely, TLS can activate and recruit effector immune cells such as T cells and B cells, ultimately establishing a fundamental structure consisting of a B-cell-rich germinal center, surrounding T-cell areas and distributed high endothelial venules (HEVs).^6^^,^^7^ The formation of TLS serves as a hallmark of localized antitumor immune activation.^8^ In multiple solid malignancies, the presence of TLS is frequently associated with favorable prognosis. Patients with HCC responsive to immunotherapy show higher TLSs enrichment in the tumor microenvironment (TME).^9^ The critical role of TLS in tumor immunity makes the characterization of TLS-related genes (TLSRGs) essential for identifying novel biomarkers for HCC therapeutic efficacy.^10^

Single-cell sequencing (scRNA-seq) is a high-throughput technique for extracting, amplifying, and analyzing single-cell gene expression profiles. Spatial transcriptomics (ST) integrates cellular gene expression with spatial coordinates in tumors, enabling in-depth study of cell-cell and cell-structure molecular interactions.^11^ The function of TLS in tumors is closely linked to their spatial distribution. Integrated single-cell and spatial transcriptomic analyses enable the discovery of novel TLS-associated biomarkers correlated with prognosis and immunotherapy response.

In this study, by integrating bulk transcriptomics, single-cell and spatial transcriptomics data from public databases and cohorts, we developed TLS-related gene signature (TLSRS) comprising 3 key TLSRGs (ACKR1, IL7R, and CCR7). We further evaluated the association of TLSRS with patients ​​​​​with HCC prognosis, TME composition, immune-related molecular features and immunotherapy response. ACKR1, CCR7, and IL7R expression patterns were further validated through bulk transcriptomic analysis, immunohistochemical (IHC), and multiplex immunofluorescence (mIF) of our cohort.

## METHODS

### Data acquisition

The Cancer Genome Atlas (TCGA, https://portal.gdc.cancer.gov) database provided bulk RNA-seq data, histopathological slides, and clinical information for 359 HCC samples. The immunophenoscore was obtained from The Cancer Immunome Atlas (TCIA, https://tcia.at).^12^ Transcriptomic and clinical data for two immunotherapy cohorts (GSE91061, n = 109; PRJEB23709, n = 91) were acquired from the TIGER platform (http://tiger.canceromics.org/). Furthermore, GEO cohorts (GSE76427, n = 115; GSE14520, n = 221) were employed for external validation to elucidate the prognostic utility of TLSRS. Single-cell data (GSE282701, n = 5) and spatial transcriptomics data (HRA000437, n = 3; GSE238264, n = 6) from tumor tissues were analyzed to validate expression patterns of TLSRS.^13^^–^^17^ Detailed characteristics of all datasets are provided in Supplemental Table S1, http://links.lww.com/HC9/C276.

Additionally, this study prospectively recruited 55 patients from Guangxi Medical University Cancer Hospital (GXMUCH cohort).^10^ They underwent hepatectomy between 2022 and 2023, with pathological confirmation of HCC. The sample collection complied with institutional ethical guidelines, the Declaration of Helsinki, and the Istanbul Protocol, with written informed consent obtained from all participants. The analysis of this cohort was approved by the Ethics Committee of Guangxi Medical University Cancer Hospital (Ethics Approval No. KY2025876). Tumor samples and matched adjacent nontumorous tissues were collected from each patient. The specific protocols for hematoxylin-eosin, IHC, and multiplex immunohistochemical were performed in accordance with previously published methodologies.^10^

Subsequently, bulk RNA sequencing was performed by Shanghai XunZhenDa Biotechnology Co., Ltd (Shanghai, China) to obtain RNA-seq data. The method for TLS assessment is described in the Supplemental Material, http://links.lww.com/HC9/C279.

### TLS abundance and survival analysis in patients with HCC

Histopathological slides of HCC acquisition from the TCGA-LIHC cohort and TLS evaluation methods were performed as described in Supplemental Material, http://links.lww.com/HC9/C279. The 359 patients with HCC were stratified into TLS (+) and TLS (−) groups. Kaplan-Meier curves for overall survival and recurrence-free survival were generated using “survminer” and “survival” R packages, with between-group statistical significance determined by the log-rank test.

### Identification of key TLSRGs

To identify novel TLSRGs, weighted correlation network analysis (WGCNA) and differential gene expression analysis were performed. As previously described, patients were stratified into TLS (+) and TLS (−) groups, and WGCNA was employed to select the most TLS-associated modules. The detailed methodology for WGCNA is provided in the Supplemental Material, http://links.lww.com/HC9/C279. Differentially expressed genes (DEGs) and module genes identified by WGCNA are provided in Supplemental Table S2, http://links.lww.com/HC9/C277. Differential gene expression analysis was then performed using the edgeR package, with DEGs defined as those meeting | log2(fold change) | > 1 and adjusted *p* value < 0.05.^18^ Volcano plots were generated using the SRplot online platform (https://www.bioinformatics.com.cn).^19^ Module genes with the highest TLS association (*p* < 0.05) overlapped with DEGs. Prognostic candidates were refined using LASSO regression to determine signature coefficients. The detailed methodology for LASSO regression is described in the Supplemental Material, http://links.lww.com/HC9/C279.

### Generation and validation of the TLSRS

The “ssGSEA” R package algorithm was employed to calculate a TLSRS score for each patient with HCC, derived from expression levels of key TLSRGs.^20^ Using the “survminer” and “survival” R packages, we determined optimal cutoff values for risk scores. Patients with scores above the dataset-specific cutoff were classified as TLSRS-high group; the rest as TLSRS-low group. To elucidate the prognostic value of the TLSRS, survival analysis was performed comparing TLSRS-high versus TLSRS-low groups and Kaplan-Meier curves were generated to demonstrate survival differences. External validation cohorts (GSE14520 and GSE76427) were incorporated to strengthen the reliability of TLSRS. Time-dependent receiver operating characteristic (ROC) curves were generated using the “timeROC” package.^21^ We further investigated the relationship between TLSRS and various clinicopathological factors by comparing these parameters across the 2 TLSRS groups. Univariate and multivariate Cox regression analyses were conducted to determine if TLSRS and clinicopathological factors can serve as independent predictors of prognosis in TCGA-LIHC cohort. Subsequently, we constructed a predictive nomogram with the use of “rms” and “regplot” packages to enable the accurate assessment of patient survival.^22^ Based on the TLS (+) and TLS (−) groups defined in the TCGA-LIHC cohort, ROC curves analyzing the predictive efficacy of the TLSRS were plotted using GraphPad Prism (v8.0).

### Gene Set Enrichment Analysis

Gene Set Enrichment Analysis (GSEA) was performed using the “org.Hs.eg.db” annotation database and “clusterProfiler” package to elucidate functional enrichment patterns and biological pathway disparities between the TLSRS-high and TLSRS-low groups. Gene set files in GMT format were obtained from the MsigDB, including Gene Ontology (GO) terms and Kyoto Encyclopedia of Genes and Genomes pathways for GSEA analysis.^23^^,^^24^ Furthermore, we employed the SRplot online platform to generate heatmaps for investigating correlations between TLSRS and 12-CK factors, TLS-50 gene signatures, and HCC molecular classes.^25^^,^^26^

### TME analysis and characterization of immune function

To evaluate the immune relevance of TLSRS, we assessed the abundance of tumor immune cells (TICs) and calculated the TME score. Specifically, we employed CIBERSORT to quantify immune cell composition.^27^ CIBERSORT analysis was performed following the official recommendations and repeated 1000 times to ensure robustness. Additionally, the ESTIMATE algorithm was employed to evaluate immune scores, stromal scores, ESTIMATE scores, and tumor mutational burden.^28^ Violin plots comparing immunophenoscore between the TLSRS-high group and TLSRS-low group were generated using the “ggpubr” package. Bar plots depicting TLSRS distribution across different immunotherapy response groups were created using the SRPlot online platform.

### Single-cell dataset processing and analysis

Data normalization was performed using the LogNormalize and ScaleData functions of “Seurat” R package, followed by identification of 2,000 variable genes with the FindVariableFeatures function. Cell clustering was conducted using the FindNeighbors function with 26 principal components (determined based on variable genes), PCA parameters set to 15, and a resolution of 0.5. After t-SNE visualization and cell type annotation, contaminating cycling cells were removed as described, resulting in 73,183 cells.^29^ For subsequent subpopulation analysis, the PCA parameters were set to 15 and a resolution of 0.2. Cell-cell communication analysis was performed using the “CellChat” package. Gene markers used to identify cell types are detailed in Supplemental Table S2, http://links.lww.com/HC9/C277.^8^^,^^16^^,^^30^^,^^31^

### Spatial transcriptomic dataset processing and analysis

We leveraged single-cell data (GSE28270) to perform cell-type spatial mapping on human HCC spatial transcriptomics data (HRA000437) using the “RCTD” package. Following data normalization with the SCTransform function in “Seurat,” ~10,000 cells—including T cells, epithelial cells, malignant cells, natural killer (NK) cells, myeloid cells, dendritic cells (DCs), hepatocytes, fibroblasts, and B cells—were analyzed in deconvolution mode to resolve spatial distribution patterns.^32^

### Experimental validation of key TLSRGs expression in HCC

HCC tissue samples were obtained from the GXMUCH cohort. To characterize the expression of key TLSRGs in HCC, we first identified TLS in HCC, followed by targeted IHC and mIF validation of these genes. The detailed procedures for IHC and mIF are described in the Supplemental Material, http://links.lww.com/HC9/C279.

### Statistical analysis

In this study, statistical analyses were performed using R software (version 4.4.0), GraphPad Prism, and various online analytical tools, including Sangerbox and SRplot. Our study employed multiple R packages, including “WGCNA,” “edgeR,” “ggplot2,” “survival,” “GSVA,” and “Seurat,” with their specific applications detailed in respective subsections. Continuous variables were compared using Student *t* test, while categorical variables were analyzed by χ^2^ test. For continuous variables that are not normally distributed, the Wilcoxon rank-sum test is used. A *p*-value < 0.05 was considered statistically significant.

## RESULT

### The presence of TLS in HCC is associated with favorable prognosis

To evaluate the prognostic significance of TLS in HCC. Patients were stratified into TLS (+) and TLS (−) groups (Supplemental Table S3, http://links.lww.com/HC9/C278). Kaplan-Meier analysis demonstrated a significant difference between these groups (*p* < 0.001), with the TLS (+) group exhibiting longer overall survival and recurrence-free survival (Figures 1A, B), consistent with our previous findings.^10^

**FIGURE 1:**
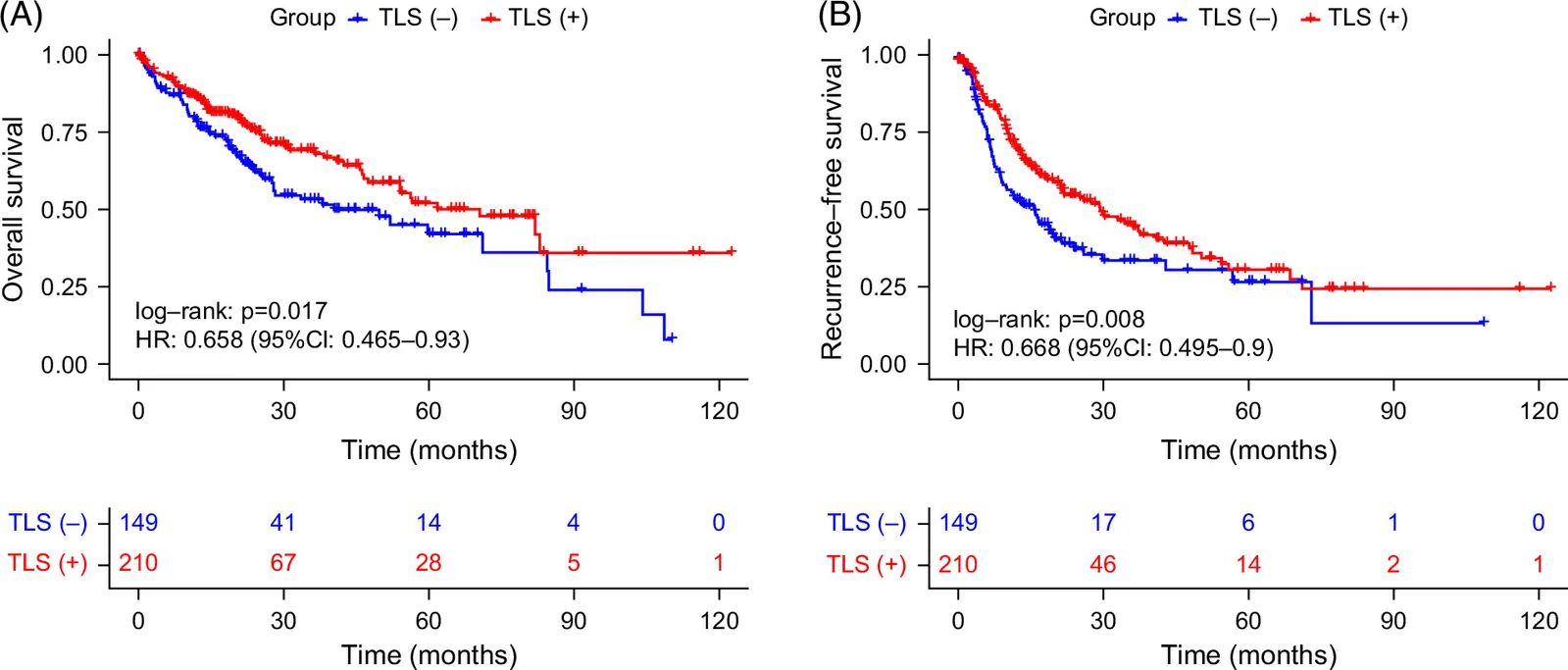
Comparison of OS (A) and RFS (B) between TLS (+) and TLS (−) groups in the TCGA-LIHC cohort. Abbreviations: OS, overall survival; RFS, recurrence-free survival; TLS, tertiary lymphoid structure.

### Establishment of a TLSRS comprising ACKR1, CCR7, and IL7R

To identify TLSRGs in HCC, we performed WGCNA analysis and identified 26 TLS-associated modules (Supplemental Figure S1, http://links.lww.com/HC9/C279). The blue and brown modules exhibited the strongest correlation with TLS (Supplemental Figure S2, http://links.lww.com/HC9/C279). Consequently, genes from these 2 modules (n = 1614) were included for subsequent analysis. Differential gene expression analysis identified 972 DEGs between the TLS (+) and TLS (−) groups (Figure 2A), including 460 upregulated genes. We intersected the upregulated genes with the module genes, resulting in 102 candidate genes (Figure 2B). Subsequent LASSO regression analysis for prognostic relevance identified a 8-gene signature (Figures 2C, D, Supplemental Table S2, http://links.lww.com/HC9/C277). Notably, ACKR1, CCR7, and IL7R belong to the cytokine or cytokine receptor. Previous studies have established that TLS formation and maintenance are critically dependent on cytokine signaling and activation.^5^^,^^6^^,^^33^ Kaplan-Meier survival analysis demonstrated that high expression levels of ACKR1, CCR7, and IL7R were significantly associated with favorable prognosis in patients with HCC (Figure 2E). We used these 3 genes to construct the TLSRS.

**FIGURE 2:**
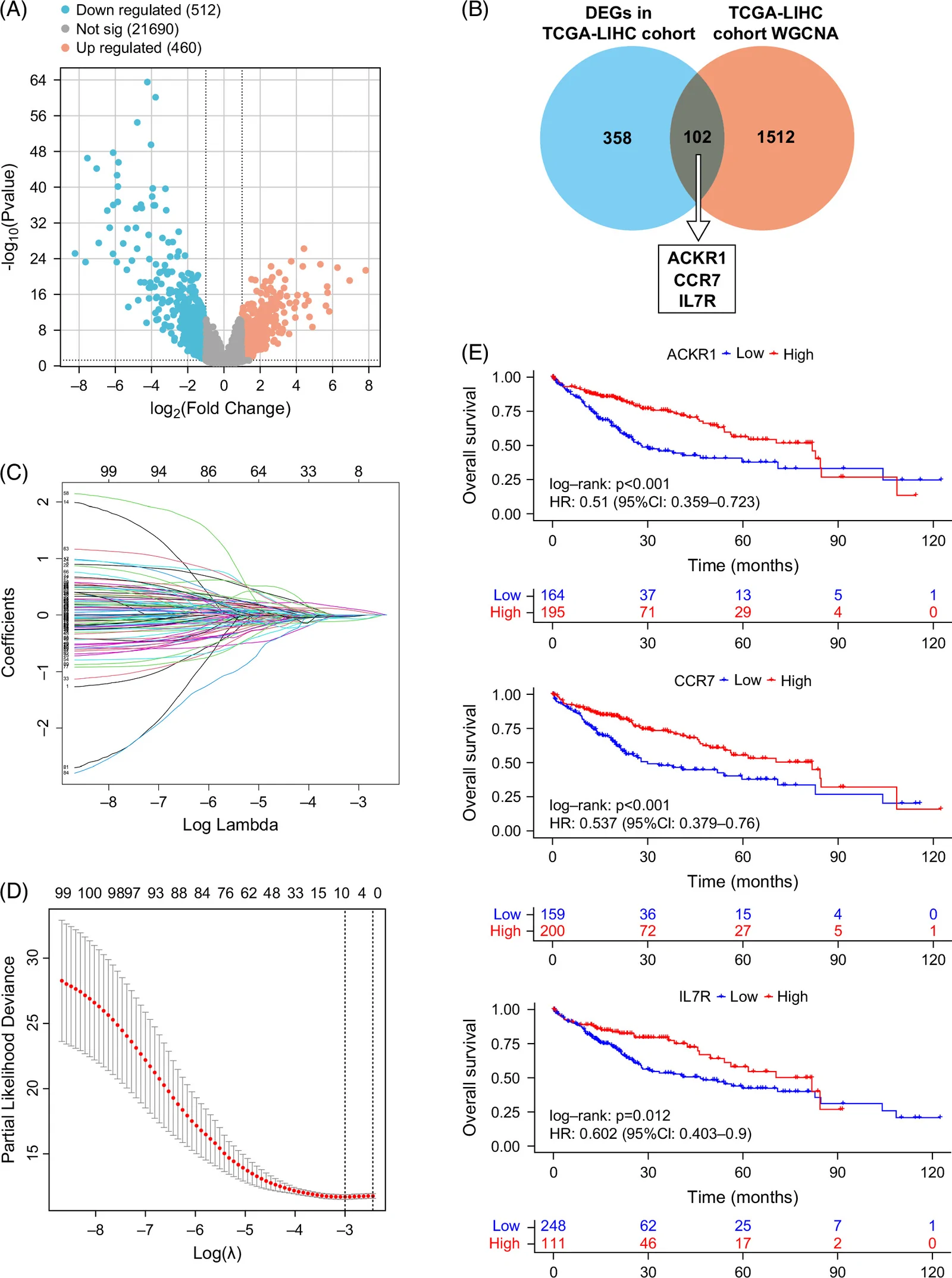
(A) Volcano plot of DEGs between TLS (+) and TLS (−) groups in the TCGA-LIHC cohort. (B) Venn diagram showing the intersection of WGCNA-derived module genes and TLS (+)-upregulated DEGs. (C) Regularization path plot from LASSO regression analysis. (D) Cross-validation error plot for LASSO coefficient selection. (E) OS comparison of ACKR1, CCR7, and IL7R expression between TLS (+) and TLS (−) groups. Abbreviations: DEGs, differentially expressed genes; OS, overall survival; TLS, tertiary lymphoid structure.

The heatmap demonstrated significantly elevated expression of both 12-CK factors^33^ and TLS-50 gene signatures^15^ of the TLSRS-high group (Supplemental Figure S3, http://links.lww.com/HC9/C279), further validating the biological relevance of TLSRS with TLS.

### TLSRS is associated with favorable prognosis in patients with HCC

To evaluate the prognostic significance of this signature, we calculated the TLSRS score for each patient with HCC in the TCGA-LIHC cohort, stratified the samples based on the optimal cutoff value (Figures 3A, C), and subsequently performed survival analysis. TLSRS-high group was significantly associated with improved overall survival and recurrence-free survival (Figures 3B, D). Consistent results were observed in the validation cohorts (GSE76427 and GSE14520) (Supplemental Figure S4, http://links.lww.com/HC9/C279). Univariate and multivariate COX analyses of this signature revealed significant associations with favorable HCC prognosis in both analyses (Figures 3E, F), indicating its independent protective role. Time-dependent ROC curves were plotted to assess the reliability of the TLSRS. The areas under the curve at different time points indicated that this TLSRS demonstrates certain prognostic predictive accuracy in both the TCGA-LIHC cohort and validation cohorts (Supplemental Figures S5A, B, http://links.lww.com/HC9/C279). Integrating independent prognostic indicators (stage and TLSRS), a predictive nomogram and calibration were created for quantitatively analyzing patient survival (Supplemental Figures S5C, D, http://links.lww.com/HC9/C279). The areas under the ROC curves indicated that TLSRS has a certain predictive ability for intratumoral TLS (Supplemental Figure S5E, http://links.lww.com/HC9/C279). Furthermore, TLSRS-high group exhibited lower-grade tumor stages, smaller tumor sizes, and reduced tumor burden (Supplemental Figure S6, http://links.lww.com/HC9/C279), demonstrating the reliability of TLSRS as a prognostic indicator for HCC.

**FIGURE 3:**
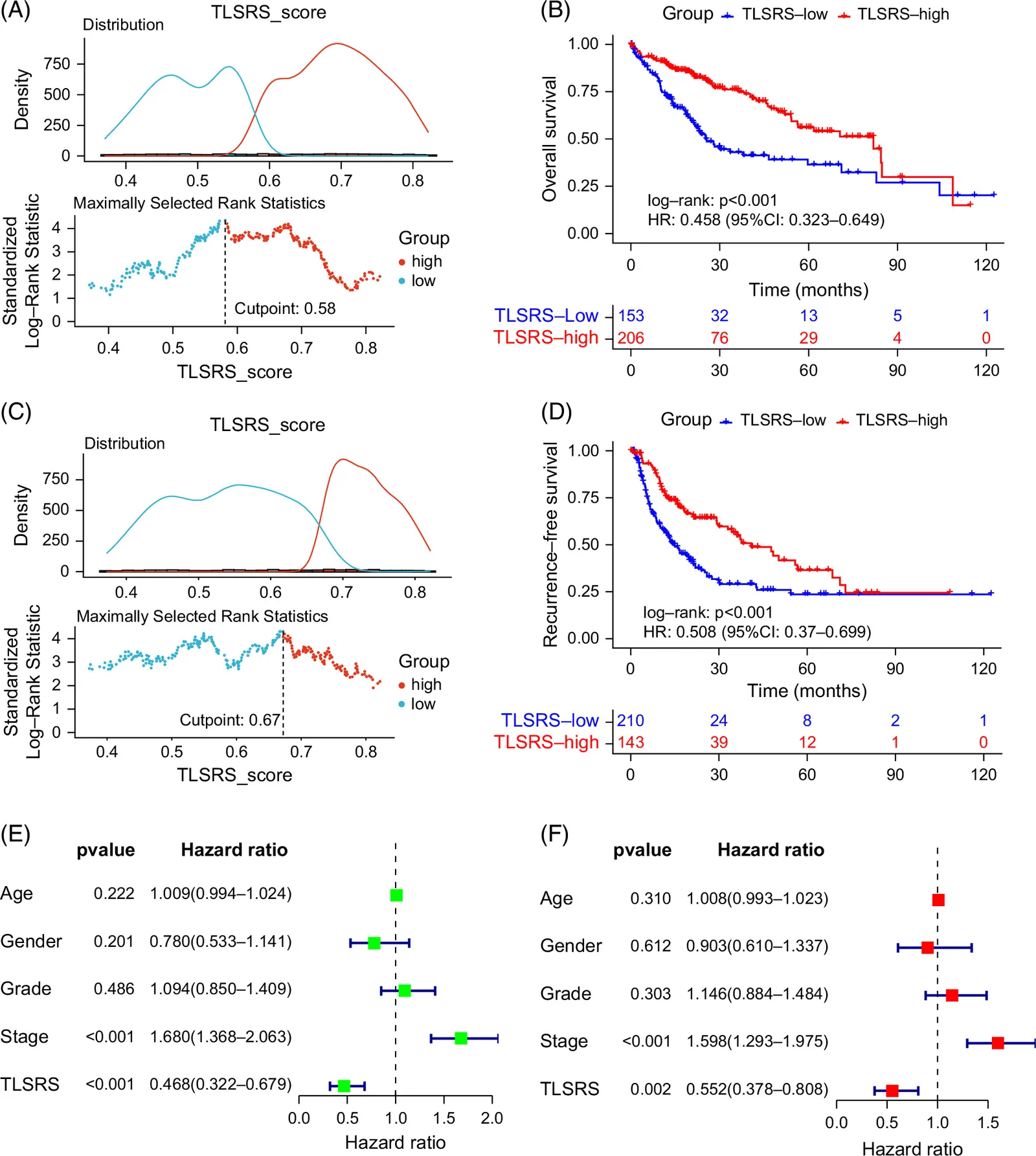
(A, C) Distribution of patients with HCC stratified by TLSRS score in the TCGA-LIHC cohort. Kaplan-Meier survival curves of OS (B) and RFS (D) comparing TLSRS high-groups and low-groups. Univariate (E) and Multivariate (F) COX regression forest plot for TLSRS. Abbreviations: OS, overall survival; RFS, recurrence-free survival; TLSRS, tertiary lymphoid structure–related gene signature.

### Immune-related gene sets were significantly enriched in the TLSRS-high group

The presence of TLS was associated with enhanced antitumor immunity, and immune-related pathways were significantly enriched in TLS-rich patients with HCC​​​​​​. GSEA results demonstrated significant enrichment of immune response-related gene sets in the TLSRS-high group, including chemokine signaling pathways, immune response, antigen receptor-mediated signaling pathways, and immune response regulation cell surface receptor signaling (Figures 4A, B). Gene sets associated with cell cycle, DNA replication, and chromosome segregation were enriched in the TLSRS-low group (Supplemental Figure S7, http://links.lww.com/HC9/C279). Furthermore, HCC inflammatory classes, PD-1 signaling signature, and expression of antigen presentation machinery genes were significantly elevated in the TLSRS-high group (Figure 4C). These findings suggest that the TLSRS-high group may exhibit enhanced immunologic activity and a more immunoreactive TME.

**FIGURE 4:**
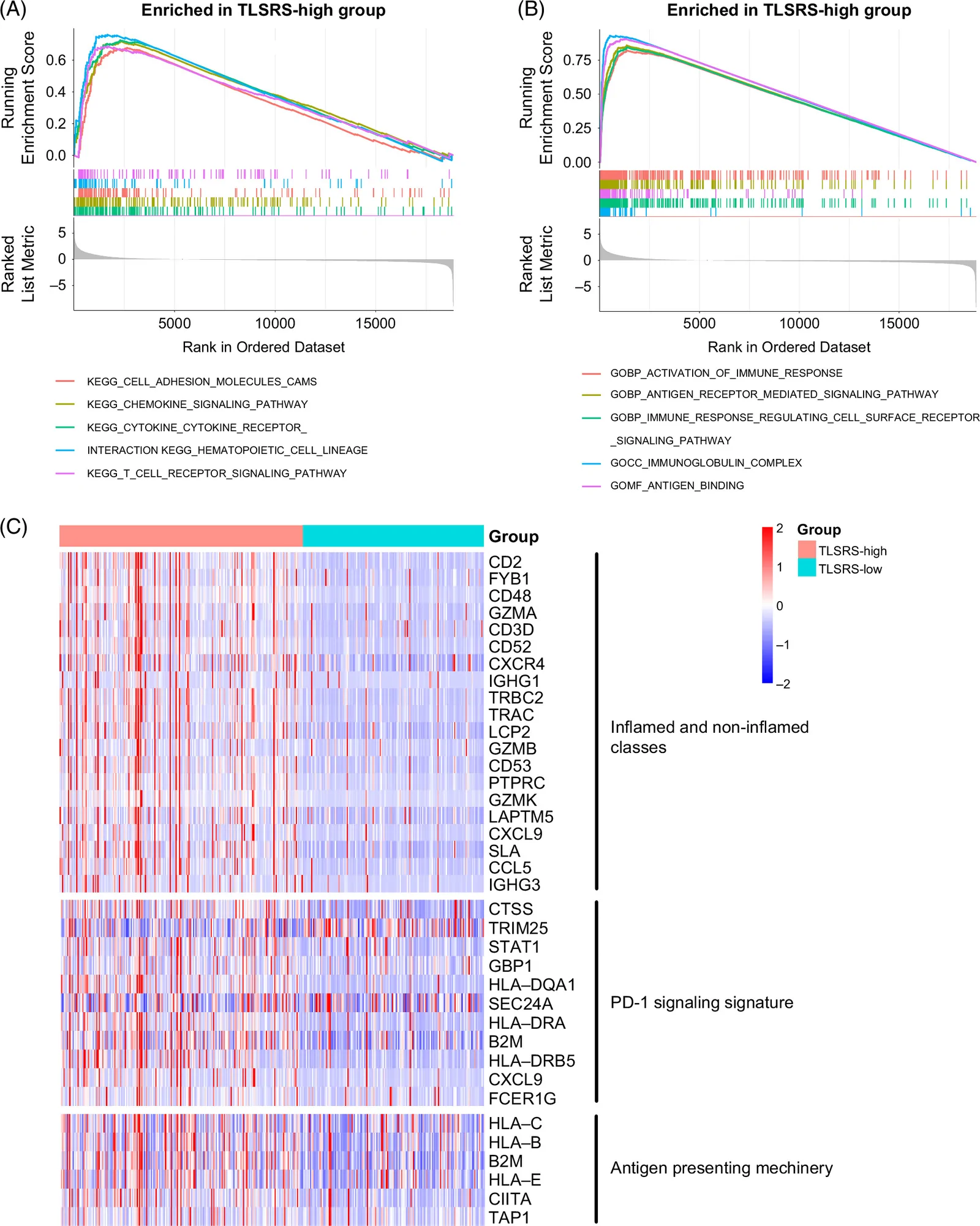
Gene Set Enrichment Analysis (GSEA) of KEGG pathways (A) and GO terms (B) in TLSRS-high groups from the TCGA-LIHC cohort. (C) Heatmap demonstrating expression profiles of inflammatory classes, PD-1 signaling signature, and antigen presentation machinery gene sets in TLSRS-high and TLSRS-low groups. Abbreviations: PD-1, programmed cell death protein 1; TLSRS, tertiary lymphoid structure–related gene signature.

### Significant differences in TME between TLSRS-high and TLSRS-low groups

The differential expression of immune response-related gene sets and immune molecular classes-associated genes between TLSRS-high and TLSRS-low groups suggests significant heterogeneity in both composition and functional states of the TME between these patients. Therefore, we employed CIBERSORT, TIMER, and ESTIMATE algorithms to evaluate TME differences. The results demonstrated significantly higher immune infiltration scores in the TLSRS-high group (Figure 5A). Compared to the TLSRS-low group, the TLSRS-high group exhibited significantly higher proportions of CD4^+^ memory T cells, CD8^+^ T cells, DCs and M1 macrophages, along with a lower proportion of M0 macrophages (Figure 5B). Furthermore, the TLSRS-high group demonstrated significantly elevated expression of most immune checkpoint-related marker genes (Figure 5C) and higher immunophenoscore (Figure 5D). In 2 independent immunotherapy cohorts (GSE91061 and PRJEB23709), a higher proportion of patients achieving partial or complete responses was observed in the TLSRS-high group (Figure 5E). Additionally, patients who achieved partial or complete responses exhibited higher expression levels of ACKR1, CCR7, and IL7R, along with elevated TLSRS scores (Supplemental Figure S8, http://links.lww.com/HC9/C279). Heatmap visualization indicates higher T-cell reactivity in the TLSRS-high group within both cohorts (Supplemental Figure S9, http://links.lww.com/HC9/C279). These results demonstrate significant TME heterogeneity between TLSRS-high and TLSRS-low groups, with higher enrichment of proinflammatory immune cell subsets in TLSRS-high tumors, which suggests an enhanced potential for clinical benefit from immune checkpoint inhibitors in this patient subgroup.

**FIGURE 5:**
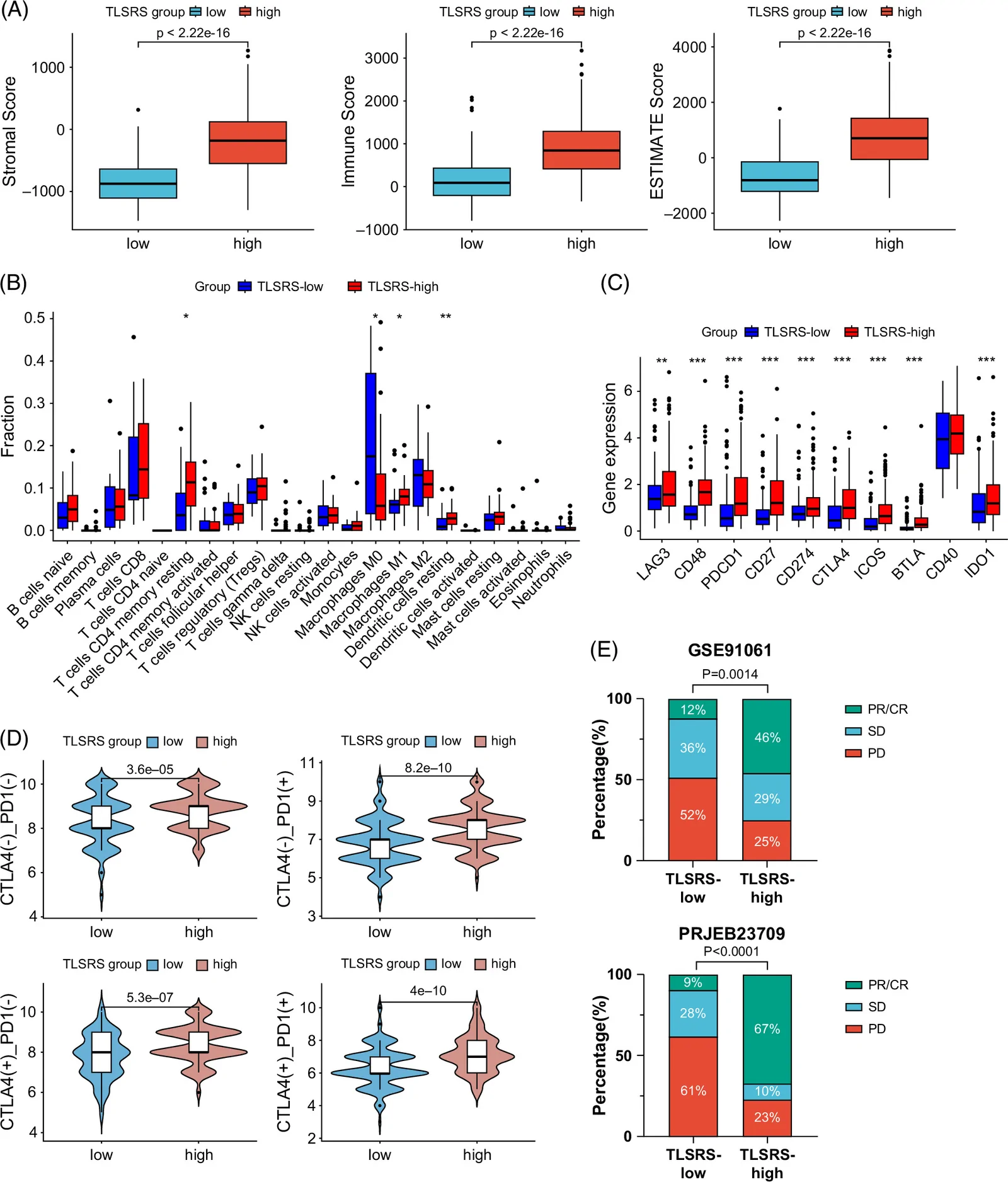
(A) Bar plots comparing ESTIMATEScore, ImmuneScore, and StromalScore between TLSRS-high and TLSRS-low groups in the TCGA-LIHC cohort. (B, C) Bar plots of differential immune cell infiltration and expression of immune checkpoint genes between TLSRS-high and TLSRS-low groups. (D) Violin plots depicting IPS stratified by TLSRS expression groups. (E) Bar graph comparing the distribution of patients with PD, SD, and PR/CR between the TLSRS-high and TLSRS-low groups in the GSE91061 and PRJEB23709 cohorts. Abbreviations: CR, complete response; IPS, immunophenoscore; PD, progressive disease; PR, partial response; SD, stable disease; TLSRS, tertiary lymphoid structure–related gene signature (*p < 0.05, **p < 0.01, ***p < 0.001).

### Single-cell analysis demonstrates elevated TLSRS expression in cell populations constituting TLS

The TLSRS-high group exhibits an immune-rich TME and demonstrates enhanced responsiveness to immunotherapy. We analyzed the GSE282701 single-cell cohort, identifying 26 distinct cell clusters through dimensionality reduction and clustering. Cell populations were annotated using established marker genes^8^^,^^16^^,^^30^^,^^31^ (Figures 6A–C; Supplemental Table S2, http://links.lww.com/HC9/C277). Our analysis revealed distinct cellular expression patterns: ACKR1 was predominantly expressed in endothelial cell clusters (clusters 1/4/14/19/25), while CCR7 showed broad expression in T cells (clusters 0/17/20), B cells (cluster 10), and DCs (cluster 6), whereas IL7R was primarily expressed in T cells (Figure 6D, Supplemental Figure S10A, http://links.lww.com/HC9/C279). Copy number variation analysis identified clusters 2, 13, 15, and 16 as malignant (Supplemental Figure S10B, http://links.lww.com/HC9/C279). To further investigate the expression of TLSRGs across refined subpopulations, T cells were categorized into CD4^+^ T cells and CD8^+^ T cells; B cells were subdivided into B cells and plasma B cells; endothelial cells were classified into endothelial cells and HEV-like endothelial cells. CD4^+^ T cells were further stratified into CCR7^+^ CD4^+^ T cells, CXCL13^+^ CD4^+^ T cells, FOXP3^+^ CD4^+^ T cells, and ANXA1^+^ CD4^+^ T cells. CD8^+^ T cells were further divided into IL32^+^ CD8^+^ T cells, IL7R^+^ CD8^+^ T cells, NFKB1^+^ CD8^+^ T cells, and PDCD1^+^ CD8^+^ T cells. DCs were classified into CD1C^+^ DCs, RGS2^+^ DCs, CLEC9A^+^ DCs, and LAMP3^+^ DCs. Fibroblasts were categorized into subpopulations, including myCAF, iCAF, protoCAF, PI16^+^, and ADH1B^+^ fibroblasts (Supplemental Figure S11, http://links.lww.com/HC9/C279). ACKR1 expression shows no significant difference between HEV-like endothelial cells and other phenotypic subsets of endothelial cells, while CCR7 expression was markedly elevated in LAMP3^+^ DCs (Supplemental Figure S12A, http://links.lww.com/HC9/C279). Cell communication analysis revealed that CCR7^+^ CD4^+^ T cells, IL7R^+^ CD4^+^ T cells, and IL7R^+^ CD8^+^ T cells exhibited extensive interactions with other immune cell subsets. Their interactions with fibroblasts and endothelial cells were comparatively weaker (Supplemental Figure S12B, http://links.lww.com/HC9/C279). These cell populations collectively form the structural and functional basis of TLS, with their coordinated expression suggesting mechanistic roles for ACKR1, CCR7, and IL7R in TLS maturation and immune coordination.

**FIGURE 6:**
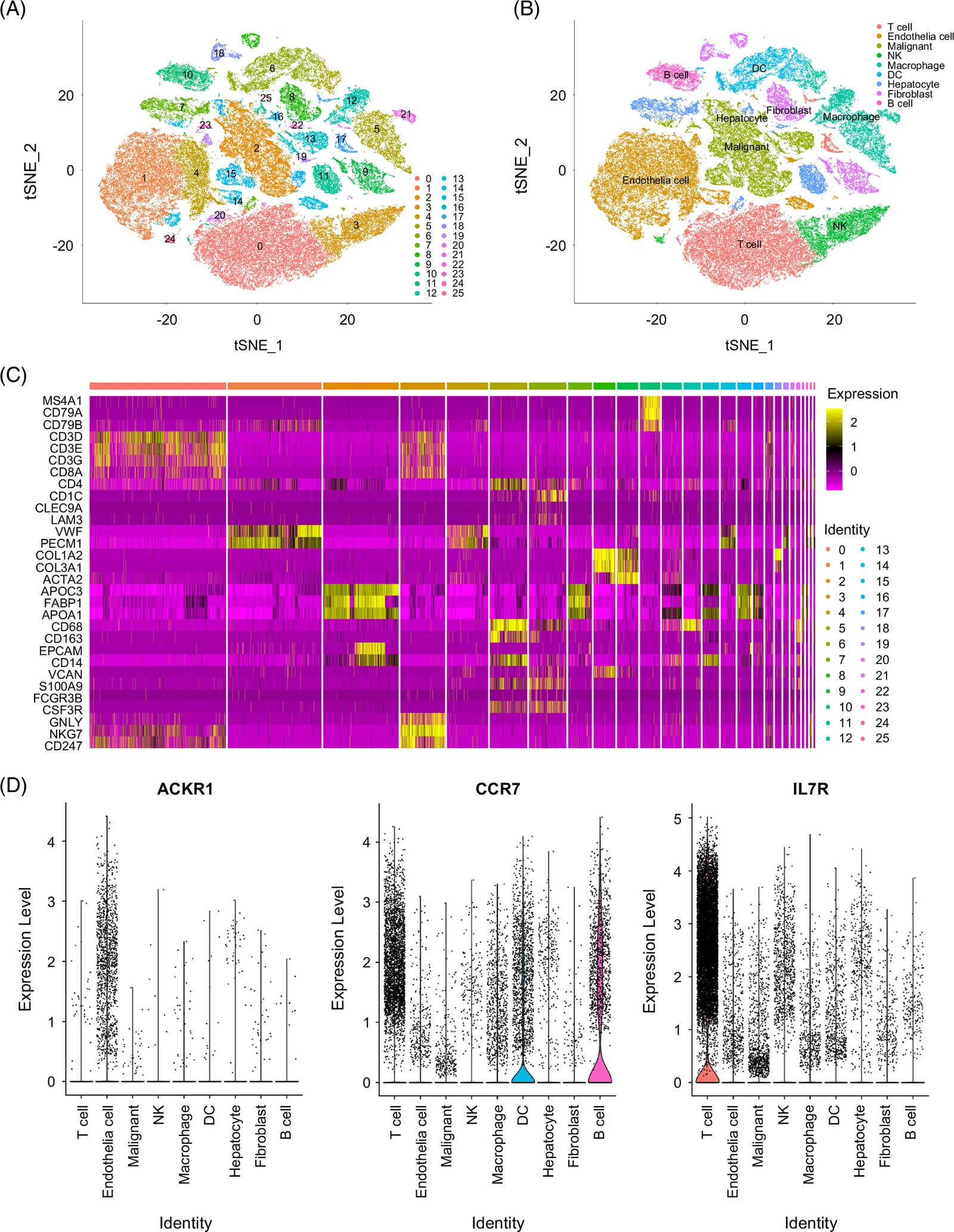
(A, B) t-SNE plot of unannotated and annotated cell clusters from GSE282701. (C) Heatmap displaying marker gene expression for each annotated cluster. (D) Violin plots show the distribution of key TLSRGs across cell subpopulations. Abbreviation: TLSRGs, tertiary lymphoid structure-related genes.

### A significant association between TLSRS and the spatial distribution of TLS

While TLSs are principally composed of immune cells, their spatial organization in HCC exhibits distinct architectural patterns (Figure 7A, Supplemental Figures S13A, D, http://links.lww.com/HC9/C279). Following dimensionality reduction and clustering, we employed the RCTD algorithm on a single-cell cohort (GSE282701) to infer the spatial distributions of endothelial cells, T cells, B cells, and DCs (Figure 7B, Supplemental Figures S13B, E, http://links.lww.com/HC9/C279). Expression profiling of ACKR1, CCR7, and IL7R revealed distinct spatial patterns: ACKR1 co-localized with endothelial cells, while CCR7 and IL7R were predominantly expressed within TLS regions and co-localized with T cells (Figure 7C, Supplemental Figures S13C, F, http://links.lww.com/HC9/C279). Moreover, analysis of spatial transcriptomics data from patients who received immunotherapy (GSE238264) revealed elevated expressions of ACKR1, CCR7, and IL7R in the responders (Supplemental Figure S14, http://links.lww.com/HC9/C279). These results further suggest that ACKR1, CCR7, and IL7R are implicated in the development of TLS and response to immunotherapy.

**FIGURE 7:**
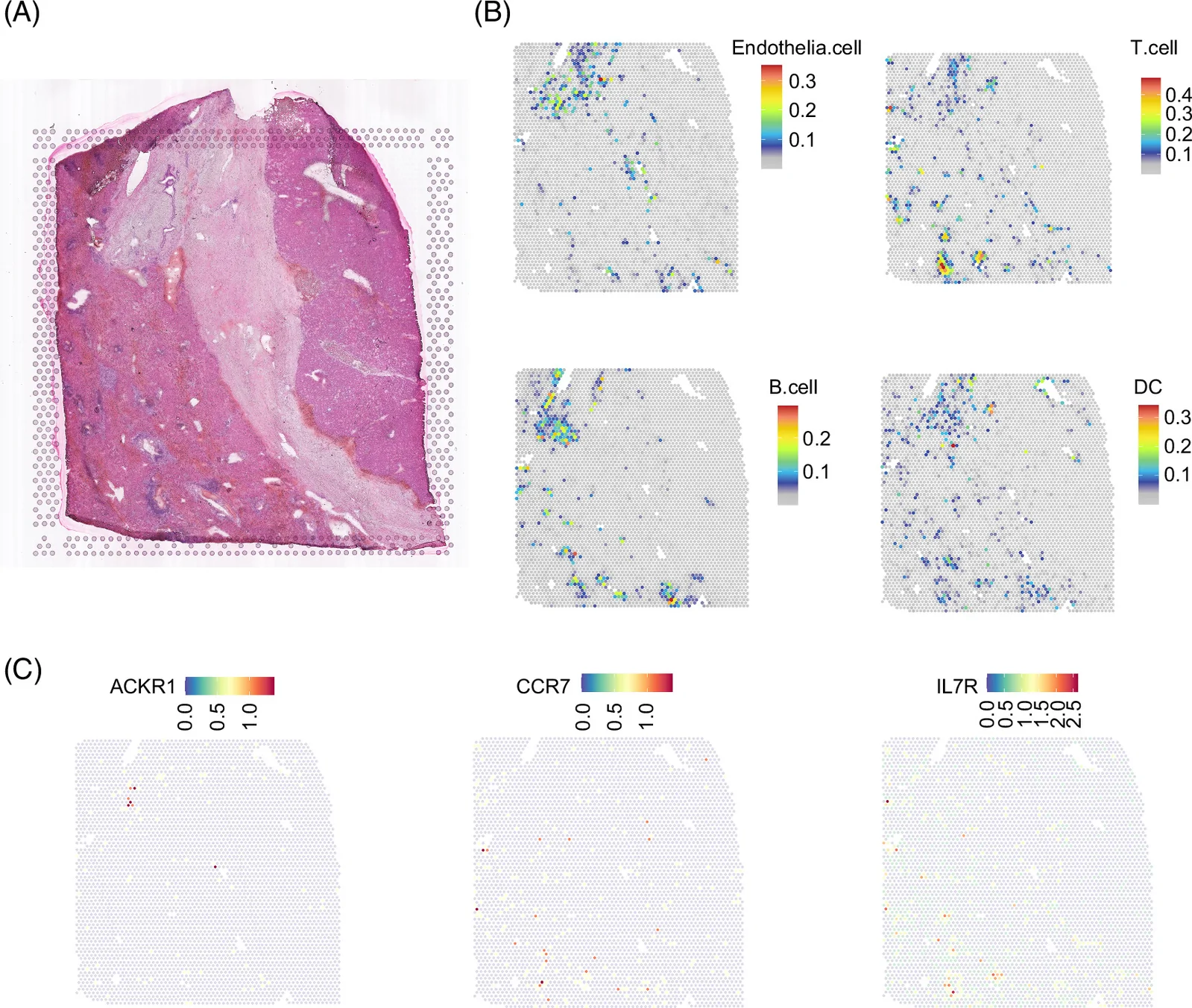
(A) Histopathological mapping of cellular clusters in HRA000437 (HCC-3L). (B) Spatial distribution of endothelial cells, T cells, B cells, and DCs. (C) Spatial expression patterns of ACKR1, CCR7, and IL7R. Abbreviation: DCs, dendritic cells.

### ACKR1, CCR7, and IL7R are expressed within TLS in the GXMUCH cohort

Spatial transcriptomics provides spatial mapping of TLSRGs critical for TLS function. We further validated the expression patterns of ACKR1, CCR7, and IL7R in the GXMUCH cohort. The IHC and multiplex immunohistochemical experimental results based on CD3^+^ T cells, CD20^+^ B cells, CD21^+^ FDCs, and CD23^+^ GC-associated markers demonstrate the presence of TLS in HCC tissues (Supplemental Figure S15, http://links.lww.com/HC9/C279).^6^ Based on the results of hematoxylin-eosin and IHC staining, we classified GXMUCH patients into TLS (+) and TLS (−) groups (Supplemental Table S3, http://links.lww.com/HC9/C278). Bulk transcriptomic sequencing analysis revealed that the expression levels of ACKR1, CCR7, and IL7R were significantly higher in the TLS (+) group (Figure 8A). IHC and mIF analysis confirmed the expression of ACKR1, CCR7, and IL7R within TLS regions in HCC tissues (Figures 8B, C).

**FIGURE 8:**
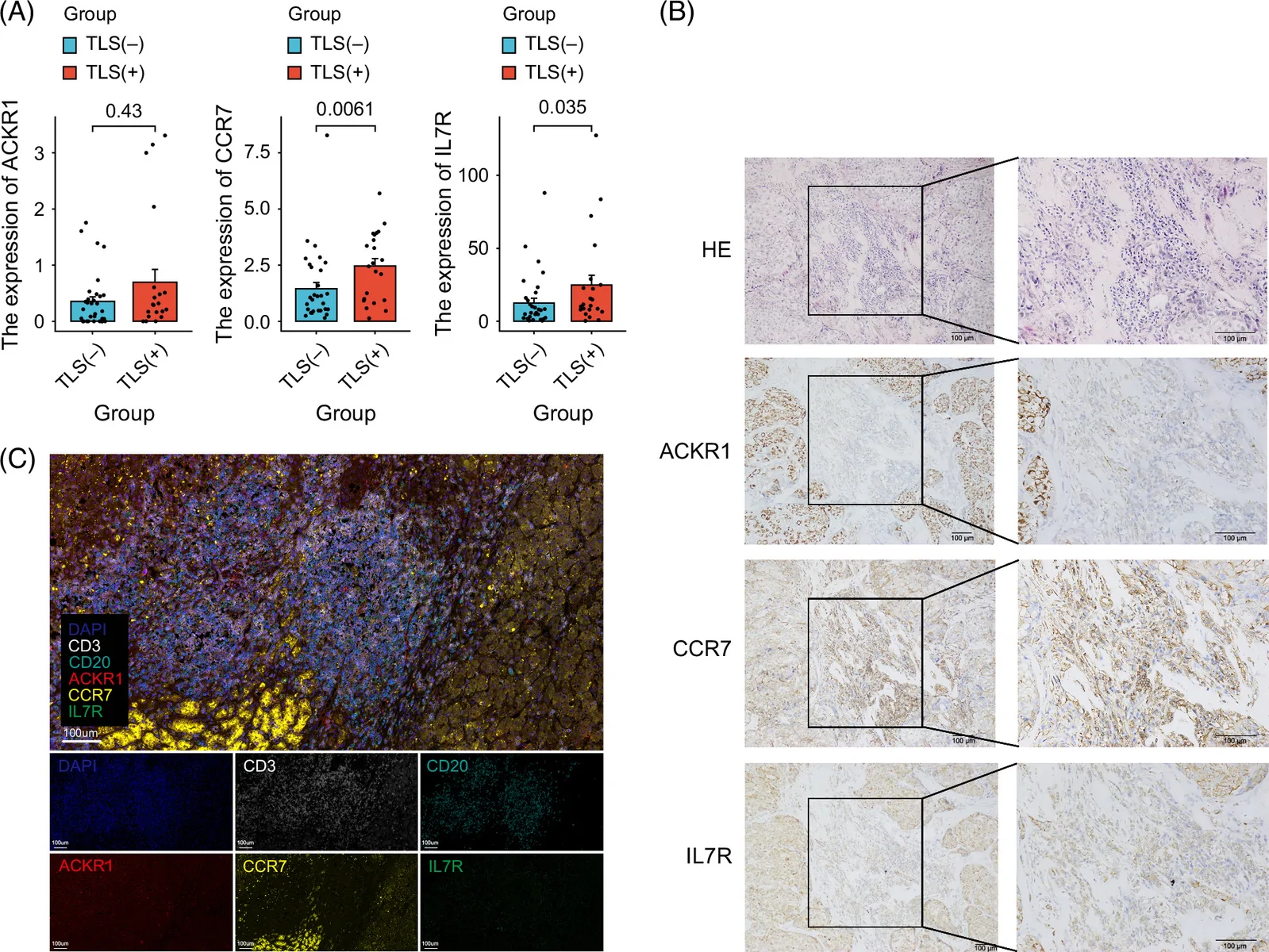
(A) Bar plots showing the gene expression levels of ACKR1, CCR7, and IL7R in TLS (+) versus TLS (−) groups from the GXMUCH cohort. (B) Representative images of HE-stained TLS in the GXMUCH cohort, along with IHC staining of ACKR1, CCR7, and IL7R. (C) Representative mIF images of CD3, CD20, ACKR1, CCR7, and IL7R in the GXMUCH cohort. Abbreviations: HE, hematoxylin-eosin; IHC, immunohistochemical; mIF, multiplex immunofluorescence.

## DISCUSSION

HCC is a major global health challenge, personalized risk-based screening is critical for its early detection and effective management. In recent years, immunotherapy for HCC has made considerable progress, though clinical responses remain unsatisfactory in a substantial proportion of patients. TME across various cancer types exhibits complex interactions among diverse immune cell populations, encompassing both immunosuppressive and immunostimulatory mechanisms,^30^ Consequently, modifying the TME can enhance the sensitivity of tumors to immunotherapy.^34^ TLS have garnered increasing attention as a key component of the TME. TLSs are primarily composed of stromal cells, B cells, T cells, macrophages, DCs, NK cells, innate lymphoid cells, and neutrophils. TLSs share structural and functional similarities with secondary lymphoid organs (SLOs).^5^^,^^6^^,^^8^ Functioning as organized aggregates of immune cells, TLS have demonstrated their ability to enhance immunotherapy efficacy in both advanced systemic therapy and neoadjuvant cohorts.^9^^,^^35^ Pretreatment biopsy specimens from immunotherapy responders consistently show significant enrichment of TLS components, particularly memory-like B cells and plasmablast-like cells.^36^ In animal models, the combination therapy of TLS induction with immune checkpoint blockade demonstrated enhanced immunotherapeutic sensitivity, generating expanded populations of effector and memory T cells.^6^ Collectively, TLS can directly potentiate immunotherapy responses or provide an immunologically favorable microenvironment. However, TLS remains challenging to assess noninvasively in clinical settings. Developing prognostic TLS-related gene signatures represents a promising research direction in HCC management.

In this study, we observed improved survival in patients with intratumoral TLS, further supporting the association between TLS and prognosis in patients with HCC. We also employed a robust key gene screening strategy, integrating bulk transcriptome-based differential gene analysis with WGCNA, identifying 102 candidate TLSRGs. Following LASSO regression, A TLS-associated prognostic and therapeutic predictive signature composed of ACKR1, CCR7, and IL7R, which serves as an independent prognostic indicator and molecular target in HCC.

While TLSRS demonstrates a certain predictive ability for the presence of TLS and is also associated with better efficacy of immunotherapy, its prognostic predictive performance is relatively weaker compared with previously published TLS-related models in HCC,^37^ potentially due to limitations in sample size.

ACKR1 is a member of the atypical chemokine receptor family and is involved in diverse biological functions, including angiogenesis and cellular chemotaxis. ACKR1 plays a critical role in tumor cell proliferation, migration, and the modulation of the TME.^38^ During TLS formation, HEV serves as critical conduits for recruiting T and B cells, playing an essential role throughout the entire TLS development process. We hypothesize that ACKR1 promotes TLS formation by facilitating HEV development. However, our identification results showed no significant difference in ACKR1 expression between HEV-like endothelial cells and other phenotypic endothelial cells. We speculate that this may be due to the lack of specific markers for HEV-like endothelial cells other than PNAd (NTAN1). CCR7 activation facilitates T-cell homing to secondary lymphoid organs while concurrently inducing DC maturation. The chemokines CCL19 and CCL21 serve as specific ligands for CCR7.^39^ Studies demonstrate that CCR7 plays a pivotal role in activating the in vivo function of FOXP3^+^CD4^+^CD25^+^ regulatory T cells (Tregs), thereby controlling the population and differentiation of immune effector cells.^40^ Within TLS, CCR7 functions as a receptor for multiple cytokines. For instance, CCR7 on T cells can bind CCL19 derived from fibroblasts to promote TLS formation,^41^ while the CXCL13-CCR7 axis recruits DCs and T cells required for TLS assembly. CXCL family chemokines may stimulate CCR7 on B cells to reduce their mobility, facilitating their retention and subsequent integration into the nascent TLS architecture.^5^ IL7R is the specific receptor for the inflammatory cytokine IL-7. It is widely expressed on lymphoid lineage cells, including NK cells, DCs, T cells, and macrophages, playing pivotal roles in the development of both T and B cells. The IL-7/IL7R interaction promotes the maturation of immature T and B cells through the JAK/STAT pathway and additionally enhances immune responses of NK cells and DCs via the PI3K/AKT pathway.^42^ During TLS development, IL-7 and its receptor (IL7R) play equally critical roles. In our study, ACKR1, CCR7, and IL7R all demonstrated strong correlations with favorable prognosis in patients with HCC and TLS formation. Building on these findings, we further investigated the associations between TLSRS and the immune microenvironment, immune cell infiltration, immune checkpoints, and immunotherapy response. Our results indicate that TLSRS correlates with enhanced immune responses in TME. Interestingly, we observed a positive correlation between TLSRS and memory T cells in tumors, providing valuable clues for further exploration of the mechanisms by which TLSRS promotes TLS formation.

Single-cell analysis revealed distinct expression patterns: ACKR1 was predominantly expressed in endothelial cells, CCR7 showed abundant expression in T cells, B cells, and DCs, while IL7R appeared to be enriched specifically in T cells—findings that are consistent with our preliminary analyses. The cell communication analysis demonstrated that CCR7^+^ CD4^+^ T cells, IL7R^+^ CD4^+^ T cells, and IL7R^+^ CD8^+^ T cells interact extensively with the cellular components of TLS. CCR7^+^ CD4^+^ T cells represent naive T cells, while IL7R^+^ CD4^+^ T cells and IL7R^+^ CD8^+^ T cells are identified as central memory T cells.^30^ These central memory T cells constitute a crucial component within the T-cell zones of TLS.^8^ CD4^+^ T cells serve as early markers of T-cell formation,^30^ whereas CXCL13^+^ CD4^+^ T cells are closely associated with TLS maturation and antitumor immune responses.^43^ Furthermore, the density of mature DCs in TLS correlates with that of CD8^+^ memory T cells, and their combined presence mediates favorable clinical outcomes in cancer patients.^44^ Spatial transcriptomic analysis demonstrated that T-cell distribution largely coincides with TLS regions. CCR7 and IL7R exhibit spatial expression patterns adjacent to TLS areas, while ACKR1 expressions localize to the periportal margins within tumor tissues. Furthermore, the expression of TLSRGs was significantly higher among the responders.

Given the pivotal roles of ACKR1, CCR7, and IL7R in TLS formation and function, we performed bulk transcriptome sequencing and IHC and mIF analysis to examine their expression patterns in the GXMUCH cohort, thereby validating our previous findings.

However, our study has several limitations. First, the GXMUCH cohort is prospectively enrolled and ongoing, both the sample size and patient follow-up information are currently insufficient. Second, spatial transcriptomic data showed incomplete concordance between immune cell markers and TLS regions, limiting our ability to investigate TLSRS across different spatial locations or maturation stages of TLS. Finally, publicly available datasets for HCC immunotherapy cohorts are currently limited. Therefore, the ability of TLSRS to evaluate TLS and immune checkpoint therapy responses requires validation in additional prospective cohorts. Orthotopic HCC models can be used to further investigate the impact of TLSRS on TLS formation and maturation, either through genetic modification of specific cell types (ACKR1 and IL7R) or via tail vein injection of CCR7.^41^

In summary, this study demonstrates that the TLSRS comprising ACKR1, CCR7, and IL7R serves as a biomarker in HCC, capable of predicting TLS presence and correlating with improved clinical outcomes and enhanced response to immunotherapy.

## Supplementary Material
