# Supplementary material for: Construction of TLS-related gene signature for predicting prognosis and immunotherapy response in hepatocellular carcinoma
Source: Hepatol Commun. 2026 Mar 26;10(4):e0925. doi: 10.1097/HC9.0000000000000925 (PMC13671625; doi:10.1097/HC9.0000000000000925)
Supplement: Supplementary file 1 [file hc9-10-e0925-s004.pdf]

Supplementary Material

Construction of TLS related gene signature for predicting prognosis and immunotherapy response in hepatocellular carcinoma

Jian-Rong Li, Li-Xin Pan, Wei Tian, Jia-Yong Su, Lei-Po Lin, Zhi-Hao Huang, Cheng-Piao Luo, Zhen-Zhen Li, Ping-Ping Guo, Liang Ma, Yi-Li Ma, Jian-Hong Zhong

Method details

1. Assessment method for TLS

After hematoxylin-eosin (HE), immunohistochemical (IHC) and multiplex immunohistochemical (mIHC) staining, Tertiary lymphoid structures (TLSs) were independently evaluated by two board-certified pathologists. Tumors with at least one TLS within the tumor margin are classified as TLS (+), while those without any TLS are classified as TLS (-). TLSs were classified into three maturity-based grades: lymphocyte aggregates (Agg), primary follicle-like structures (FL1), and secondary follicle-like structures (FL2).[1] In cases of discrepant assessments, a third senior pathologist was consulted for definitive evaluation.

2. The detailed methodology of WGCNA

Weighted gene co-expression network analysis (WGCNA) was performed using the WGCNA R package.[2] Prior to network construction, gene expression data were preprocessed to remove low-variance genes (filtering criteria: genes with a variance below the 25th percentile across all samples were excluded). After generating the clustering tree using the sampleTree function, outlier samples were excluded with the cutreeStatic function, where the parameter minSize was set to 10.

To construct the weighted co-expression network, the following key parameters were applied:

(1) A soft-thresholding power ($\beta = 9$) was determined by analyzing the scale-free topology fit index (aiming for a fit index >0.9) and mean connectivity, ensuring the network approximate scale-free properties.
(2) Pairwise Pearson correlations between genes were calculated to generate an adjacency matrix, which was then transformed into a topological overlap matrix (TOM) to minimize the effects of noise and spurious correlations.
(3) Gene modules were identified using the dynamic tree cut algorithm with minimum module size set to 100 genes, and a merging threshold of 0.25 was used to merge highly similar modules.

Module-trait relationships were evaluated by calculating the correlation between module eigengenes (MEs, the first principal component of each module) and clinical traits, with significance determined by Student's t-test. Hub genes within key modules (those significantly associated with target traits, $p < 0.05$) were defined as genes with high intramodular connectivity.

3. LASSO regression analysis

Least Absolute Shrinkage and Selection Operator (LASSO) regression was performed using the glmnet R package to select key predictive genes and construct a parsimonious predictive model.[3] Merge duplicate genes and filter out low-expression genes. The family parameter was set to "cox" (Cox-LASSO).

Regularization strength (λ) was optimized via 10-fold cross-validation using the cv.glmnet function. The optimal λ (λ_{opt}) was defined as the value that minimized the cross-validation error. A secondary threshold (λ_{1se}), representing the largest λ with an error within 1 standard error of the minimum, was also considered to ensure model stability. The penalty factor (α) was set to 1, confirming strict LASSO regression (as opposed to elastic net, where $0 < \alpha < 1$). Genes with non-zero coefficients at λ_{opt} were identified as "selected genes" and retained for downstream analyses.

4. Detailed procedures for IHC

Paraffin sections are dewaxed with xylene and rehydrated with gradient ethanol. And then antigen retrieval is performed by microwave heating in EDTA or citrate buffer, followed by PBS washing after cooling. Add BSA and incubate at room temperature for 30 minutes. HCC tissue sections were incubated overnight at 4°C with primary antibodies against ACKR1 (Sabbiotech, 56458-1, 1:200), CCR7 (Abcam, ab253187, 1:500), and IL7R (Abcam, ab259806, 1:500), followed by 1h incubation with enhanced enzyme-labeled polymer conjugate of goat anti-mouse/rabbit IgG (ZSGB-Bio, Beijing, China) at room temperature. Antigen-antibody complexes were visualized using DAB chromogen, and sections were counterstained with hematoxylin.

5. Detailed procedures for multiplex immunofluorescence (mIF)

First, sample pretreatment is performed. Paraffin sections are sequentially dewaxed with environmentally friendly dewaxing solution, rehydrated to distilled water through pure ethanol, and then placed in EDTA for antigen retrieval by microwave heating. After natural cooling, the sections are washed 3 times with PBS for 5 minutes each time. After slightly drying, the tissue area is circled with a histochemical pen, and blocking solution (BSA) is added for blocking at room temperature for 30 minutes to reduce non-specific binding. Next, antibody incubation is carried out: after discarding the blocking solution, add the diluted primary antibody, place the sections in a humid chamber and incubate at 4°C overnight. On the next day, wash 3 times with PBS to remove unbound primary antibody, then add HRP-conjugated secondary antibody and incubate at room temperature for 50 minutes, and repeat the washing step after incubation. Then signal amplification is performed: add TSA working solution and incubate at room temperature for 10-15 minutes to enhance the fluorescent signal. Wash and elute the antibody by immunostaining antibody striping buffer to avoid cross-reaction. Repeat the above antibody incubation and signal amplification steps, replacing the primary antibody against different targets and corresponding fluorescent dyes each time until all targets are labeled. Finally, nuclear staining and mounting are conducted: add DAPI staining solution and incubate in the dark at room temperature for 10 minutes to counterstain cell nuclei. After washing, mount the sections with anti-fade mounting medium. In the imaging analysis stage, images are collected using a multispectral fluorescence microscope. Primary antibodies for mIF against CD3

(GB12014), CD20 (GB155721), and CCR7 (GB11502) were purchased from Servicebio (Wuhan, China), ACKR1 (55185-1-AP), and IL7R (17626-1-AP) were purchased from Proteintech (Wuhan, China).

Supplementary Figure 1. (A) Cluster dendrogram from the TCGA-LIHC cohort. (B) Scale independence and mean connectivity plots from WGCNA analysis.

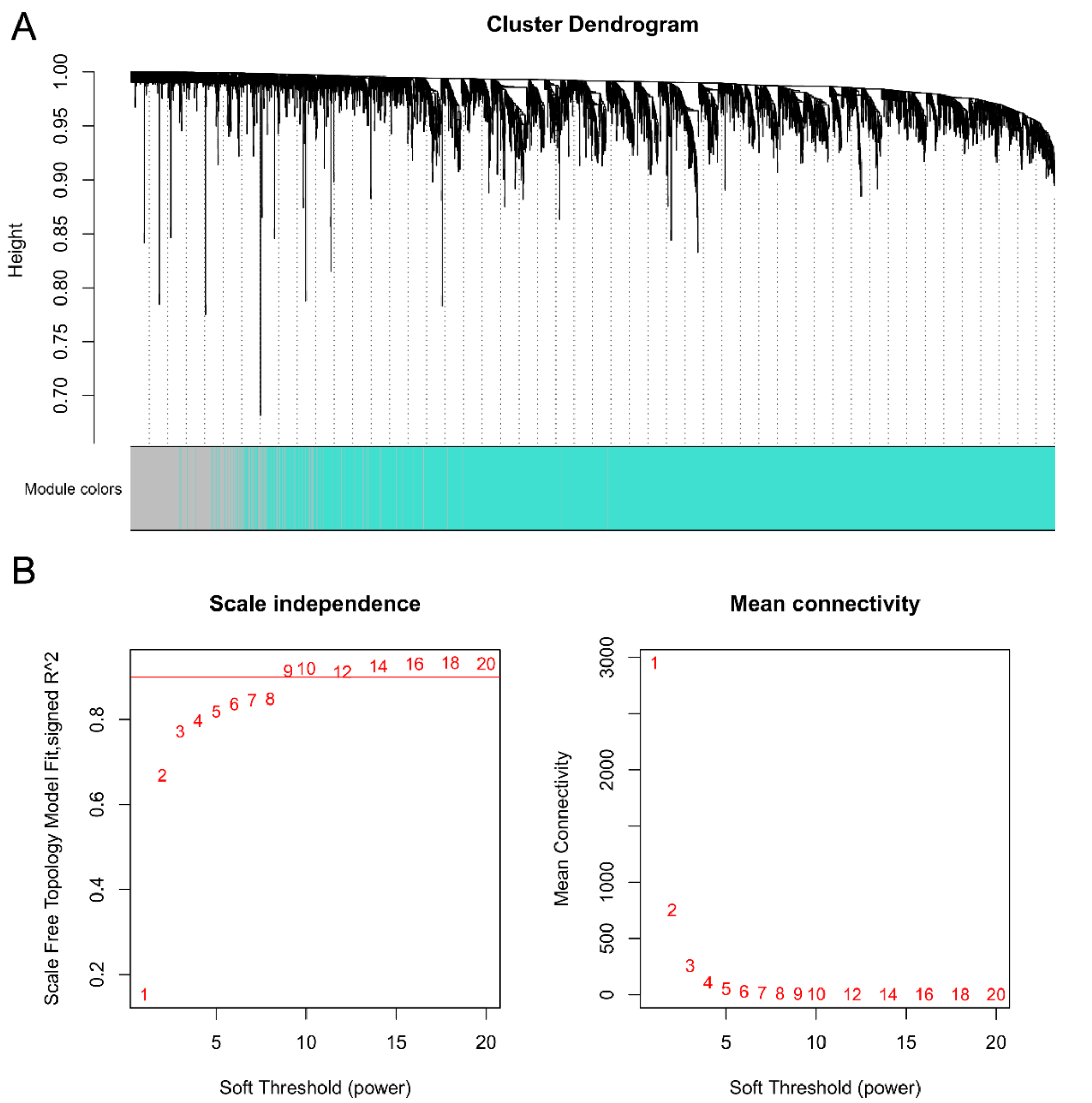

Supplementary Figure 2. Heatmap of module-trait correlations from WGCNA analysis in the TCGA-LIHC cohort. TLS, tertiary lymphoid structure.

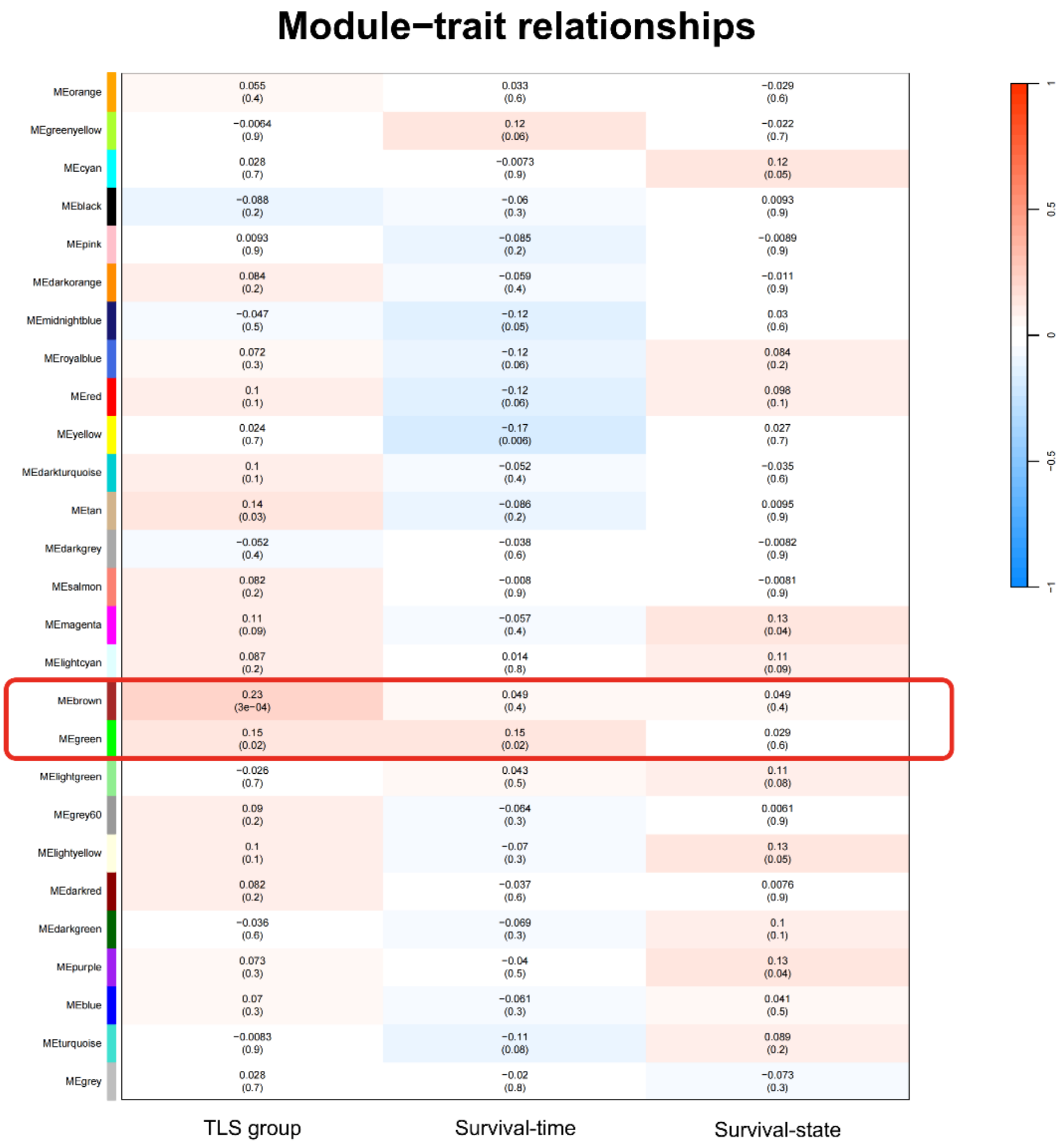

Supplementary Figure 3. Heatmap depicting expression patterns of 12-CK factors (A) and TLS-50 gene signatures (B) in TLSRS-high versus TLSRS-low groups (TCGA-LIHC cohort). TLSRS, tertiary lymphoid structure-related gene signature.

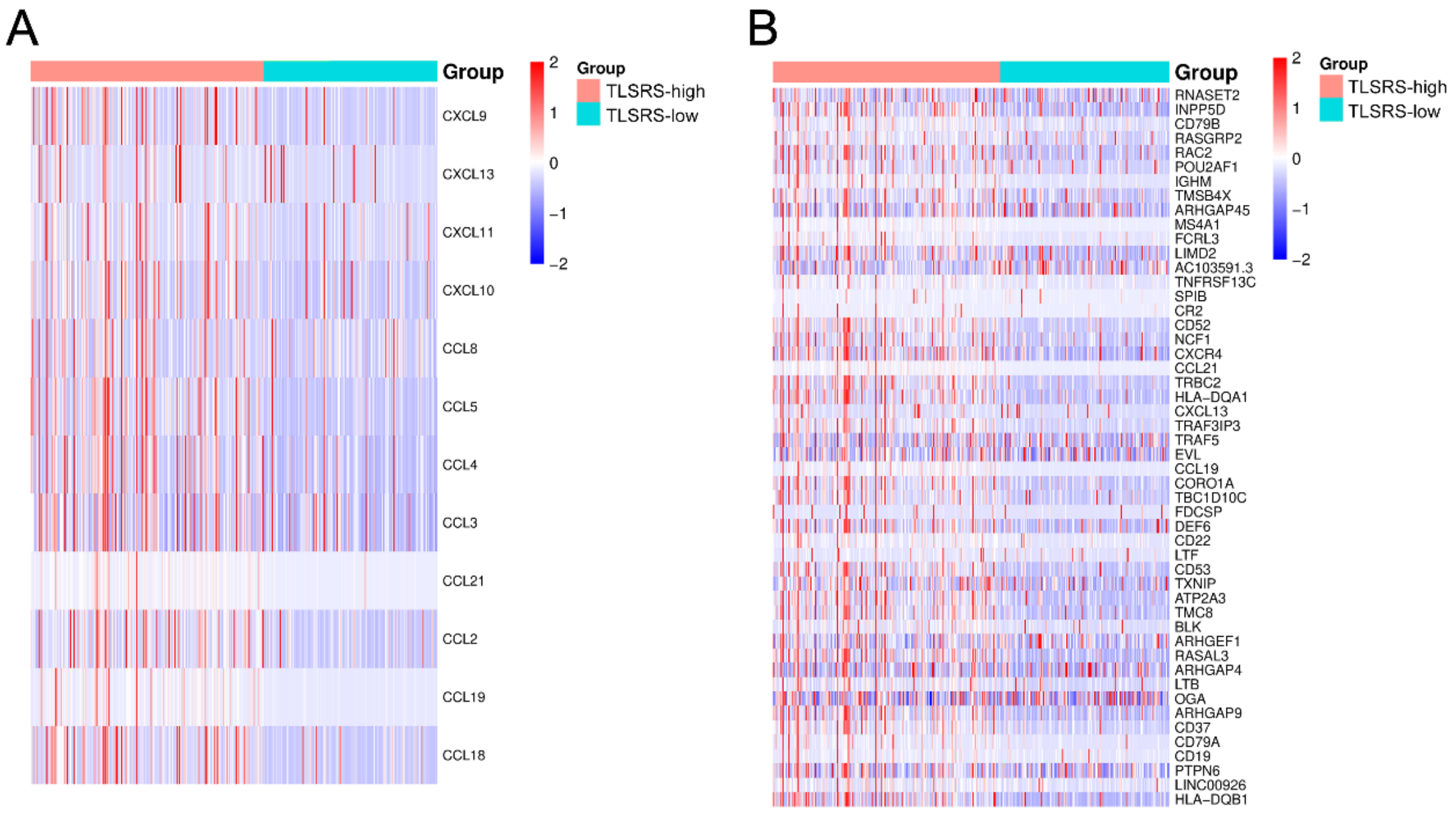

Supplementary Figure 4. Distribution and survival curves of OS and RFS with TLSRS-high and TLSRS-low groups in the GSE76427 cohort (A) and GSE14520 cohort (B). TLSRS, tertiary lymphoid structure-related gene signature; HR, hazard ratio; OS, overall survival; RFS, recurrence-free survival.

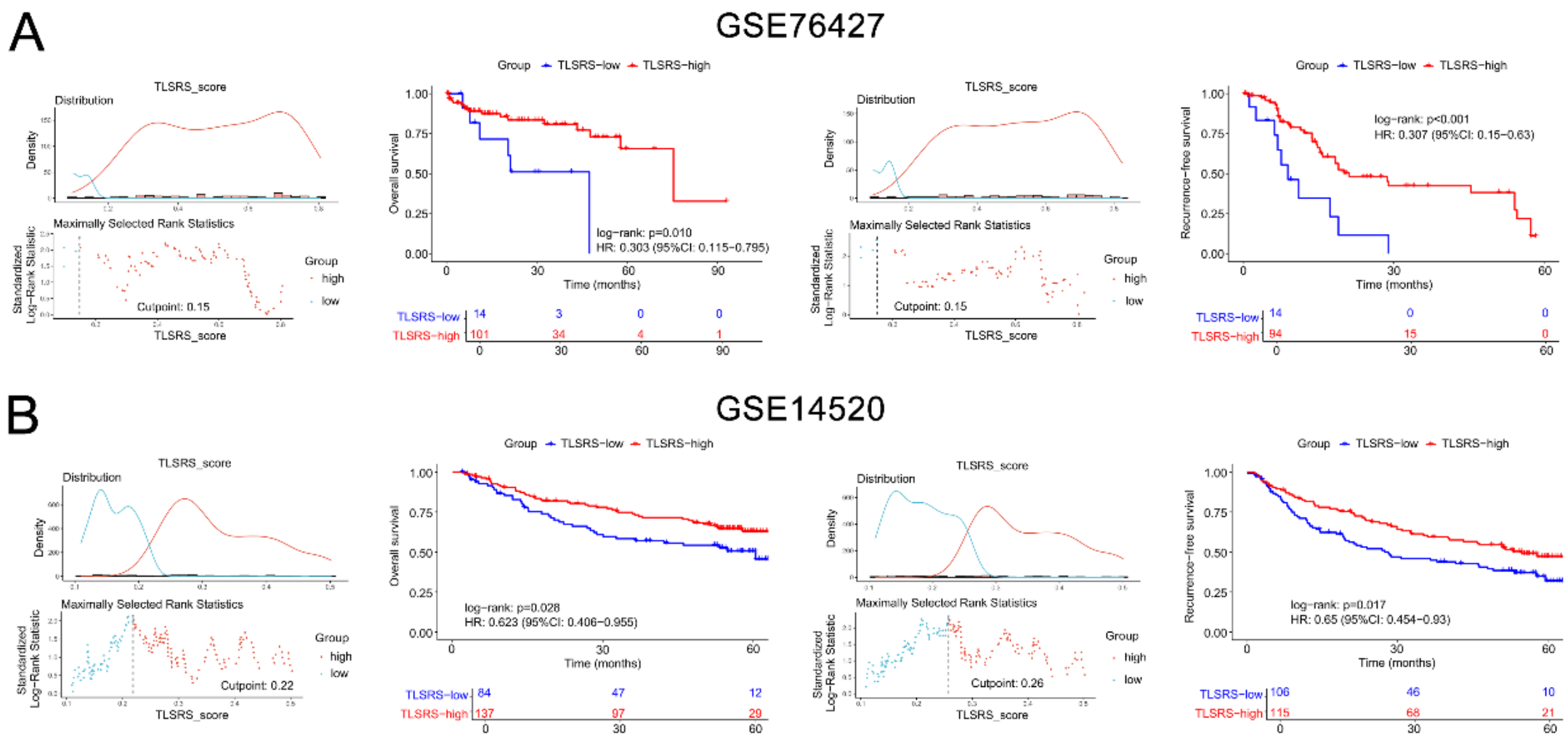

Supplementary Figure 5. (A, B) The time-dependent ROC curves of TLSRS in the TCGA-LIHC cohort and GSE76427 cohorts. (C, D) A predictive nomogram model of TLSRS. (E) ROC curve for predicting TLS presence/absence using TLSRS in the TCGA-LIHC cohort. TLS, tertiary lymphoid structure; TLSRS, tertiary lymphoid structure-related gene signature.

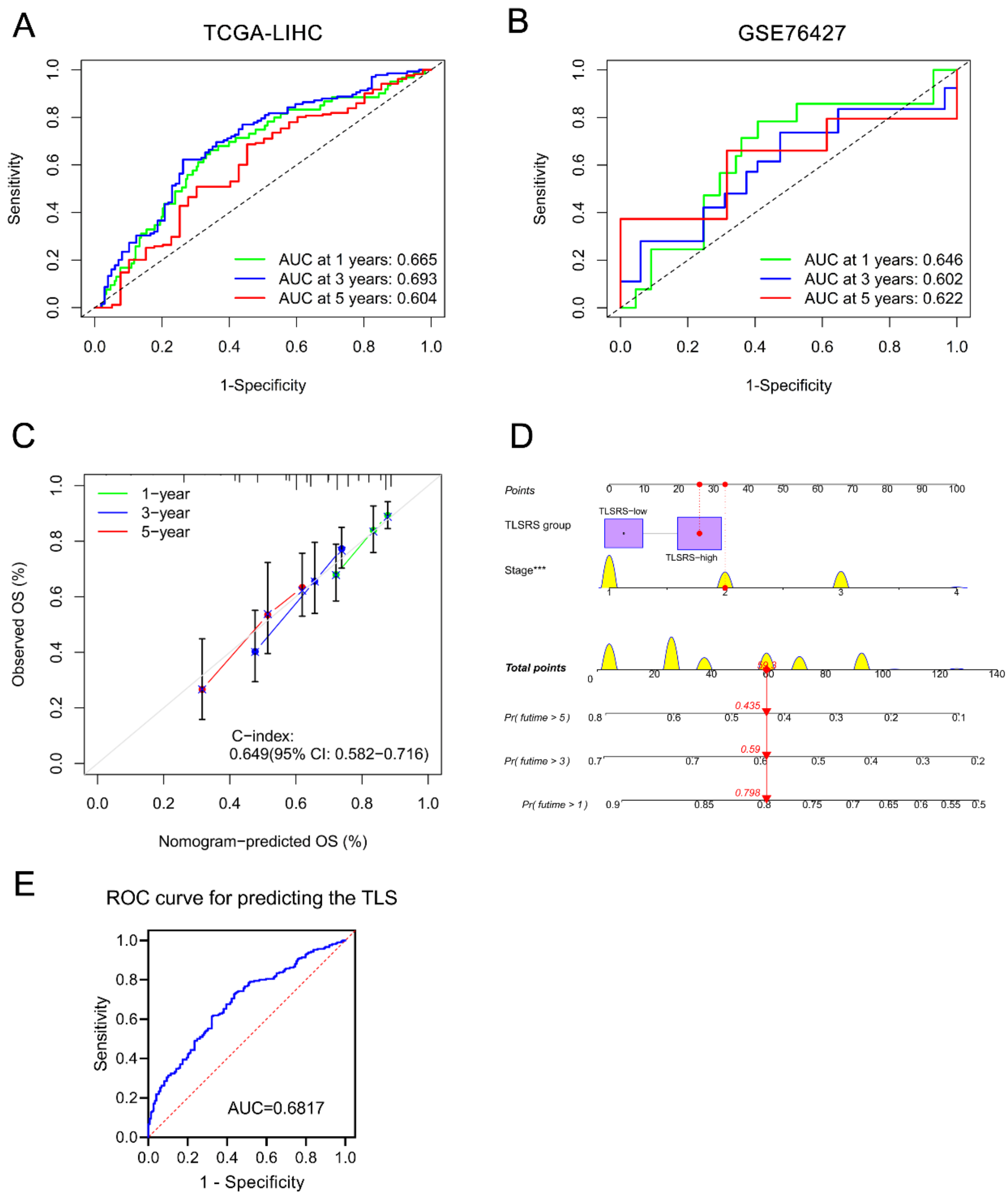

Supplementary Figure 6. (A) Stacked bar plots showing the percentage distribution of clinical traits between TLSRS-high and TLSRS-low groups; (B) Bar plot comparing tumor burden between TLSRS-high and TLSRS-low groups; (C) Scatter plot analyzing the correlation between TLSRS score and tumor burden. TLSRS, tertiary lymphoid structure-related gene signature; T, tumor.

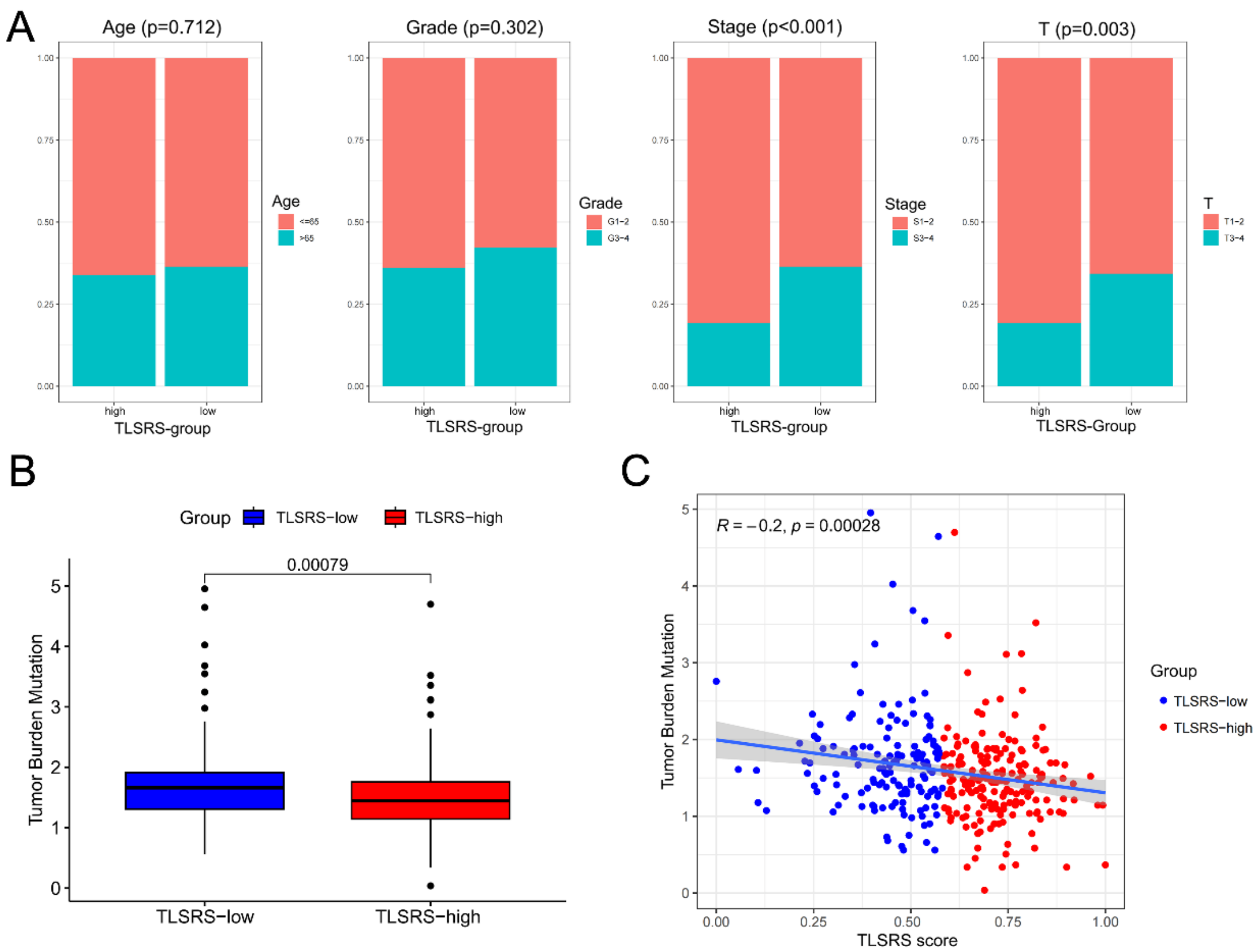

Supplementary Figure 7. Gene Set Enrichment Analysis (GSEA) of KEGG pathways (A) and GO terms (B) in TLSRS-low groups from the TCGA-LIHC cohort. TLSRS, tertiary lymphoid structure-related gene signature.

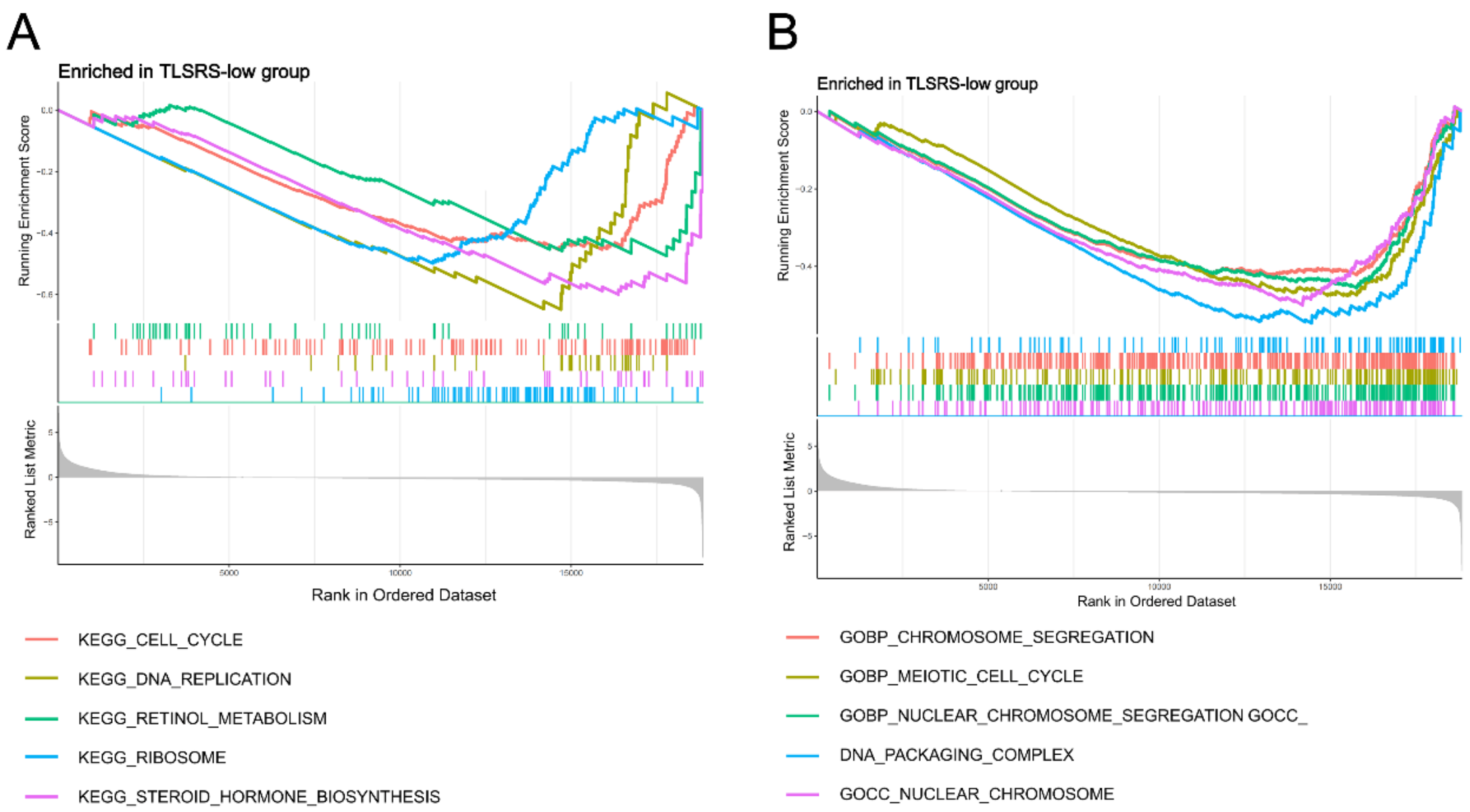

Supplementary Figure 8. (A-B) Expression profiles of ACKR1, CCR7, and IL7R in two immunotherapy cohorts (GSE91061 and PRJEB23709); (C-D) Distribution of TLSRS scores in two immunotherapy cohorts (GSE91061 and PRJEB23709). TLSRS: tertiary lymphoid structure-related gene signature; CR, complete response; PR, partial response; SD, stable disease; PD, progressive disease.

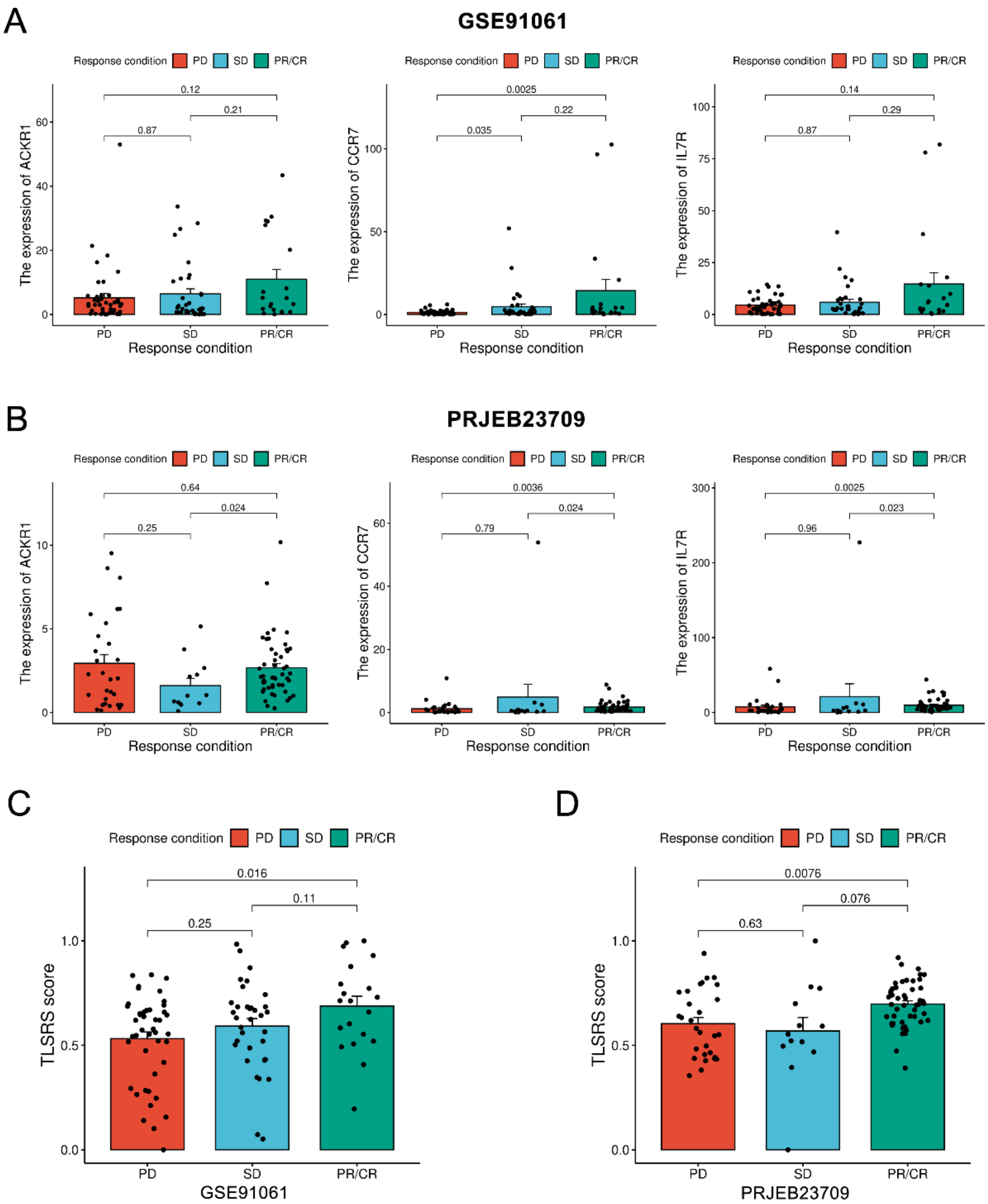

Supplementary Figure 9. Comparison of T-cell gene signatures between the TLS-high and TLS-low groups across different immunotherapy cohorts (GSE91061 and PRJEB23709).

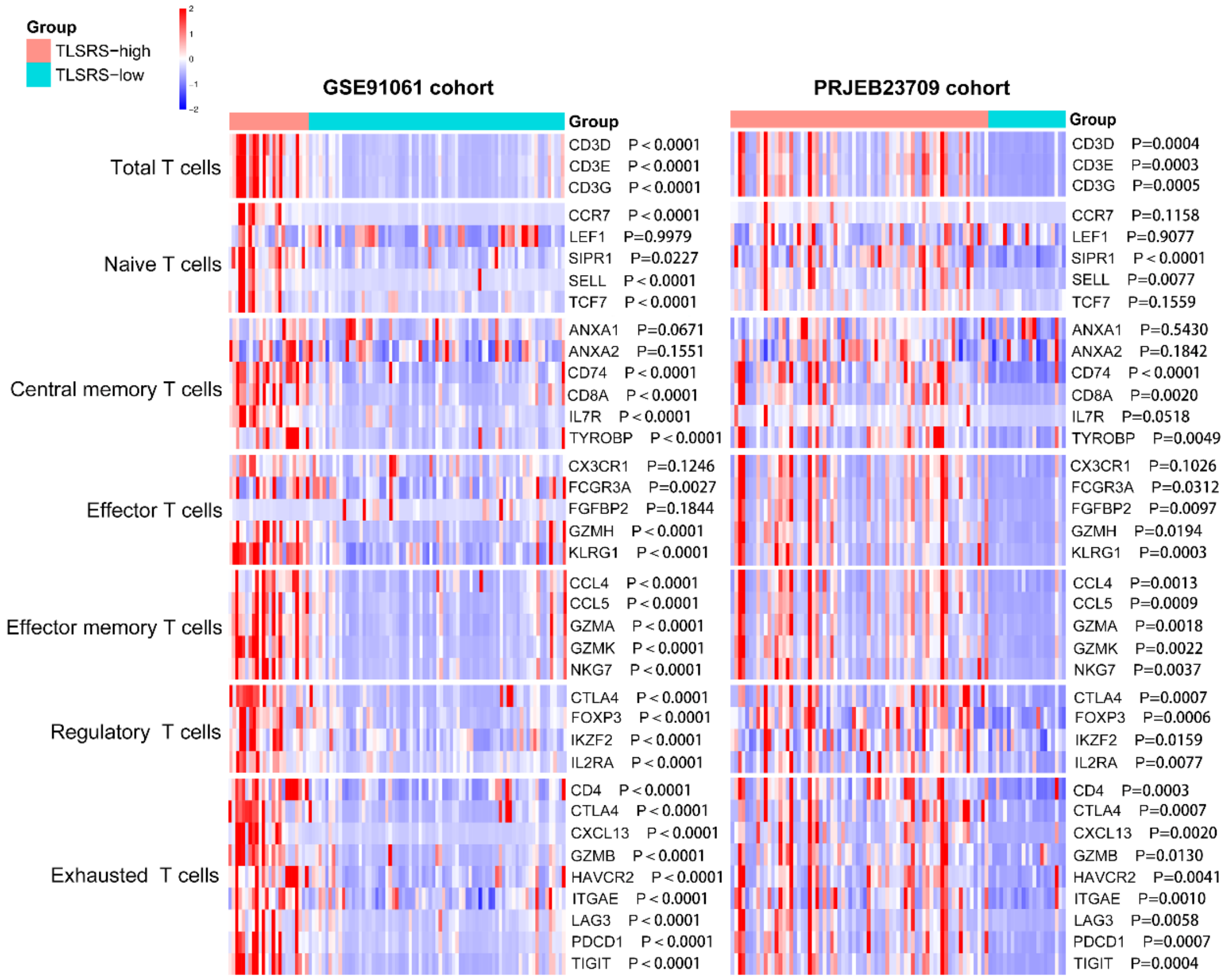

Supplementary Figure 10. (A) Expression distribution of ACKR1, CCR7, and IL7R across cellular subpopulations; (B) Copy number variation (CNV) analysis in selected malignant cell subpopulations.

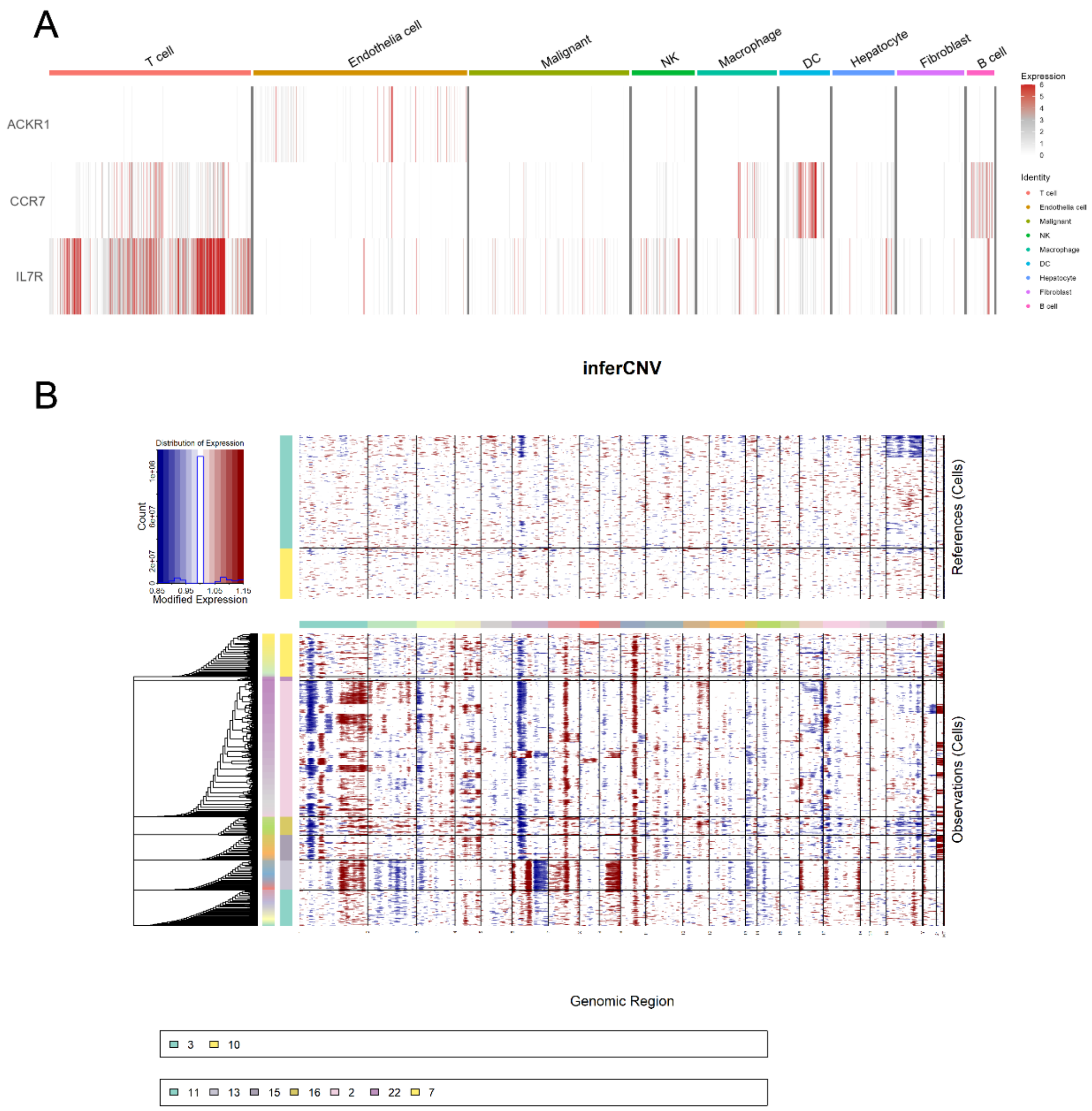

Supplementary Figure 11. Dot plot of cellular subpopulations for T cells (A), B cells (B), endothelial cells (C), $CD4^{+}$T cells (D), $CD8^{+}$T cells (E), DCs (F), and fibroblasts (G) subsets. DCs, dendritic cells.

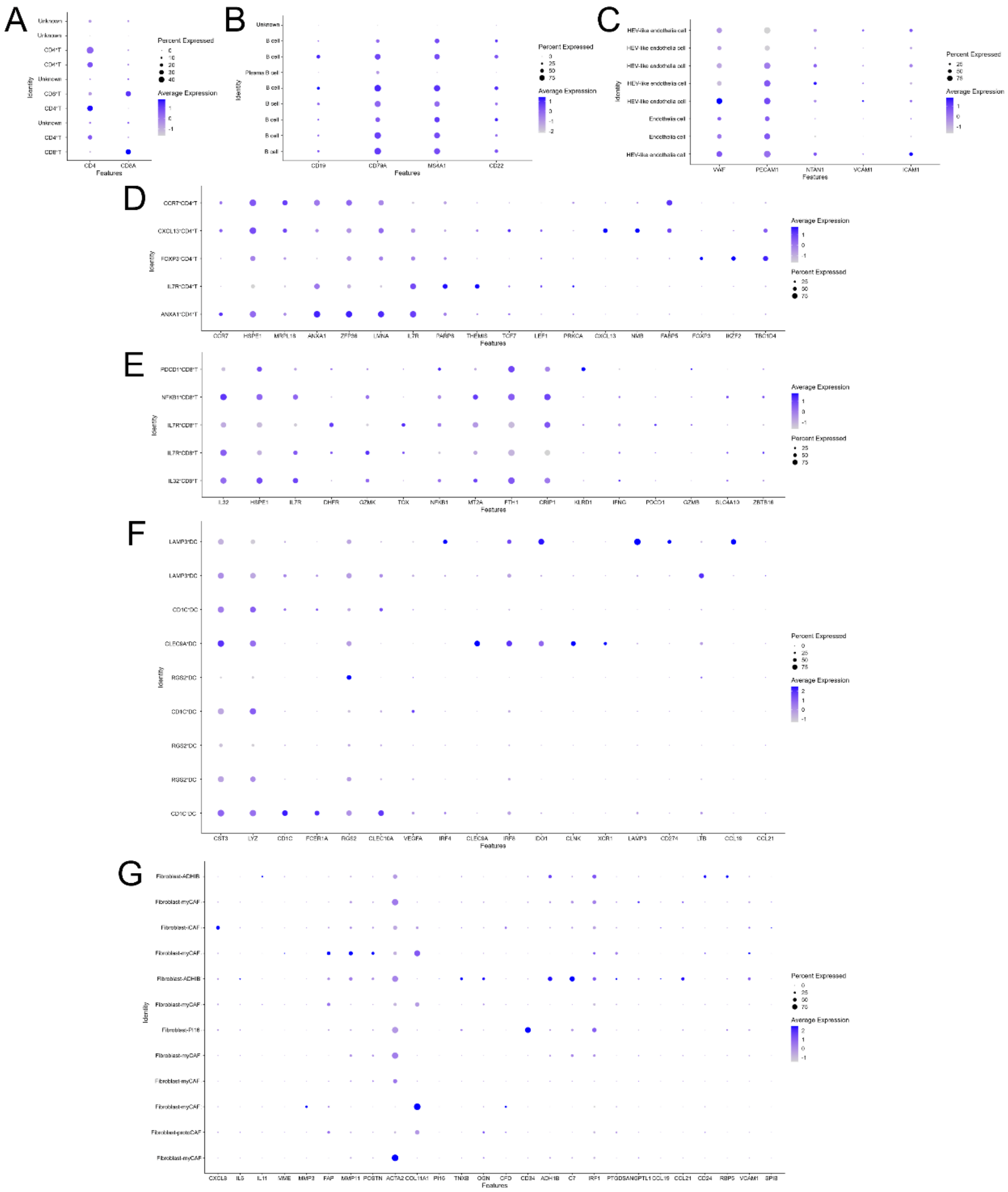

Supplementary Figure 12. Violin plots show the expression of ACKR1, CCR7, and IL7R in distinct cellular subpopulations (A) and the pattern of interaction strength/probability between CCR7$^+$CD4$^+$ T cells, IL7R$^+$CD4$^+$ T cells, and IL7R$^+$CD8$^+$ T cells with distinct subsets of T cells, B cells, DCs, and fibroblasts (B). DCs, dendritic cells.

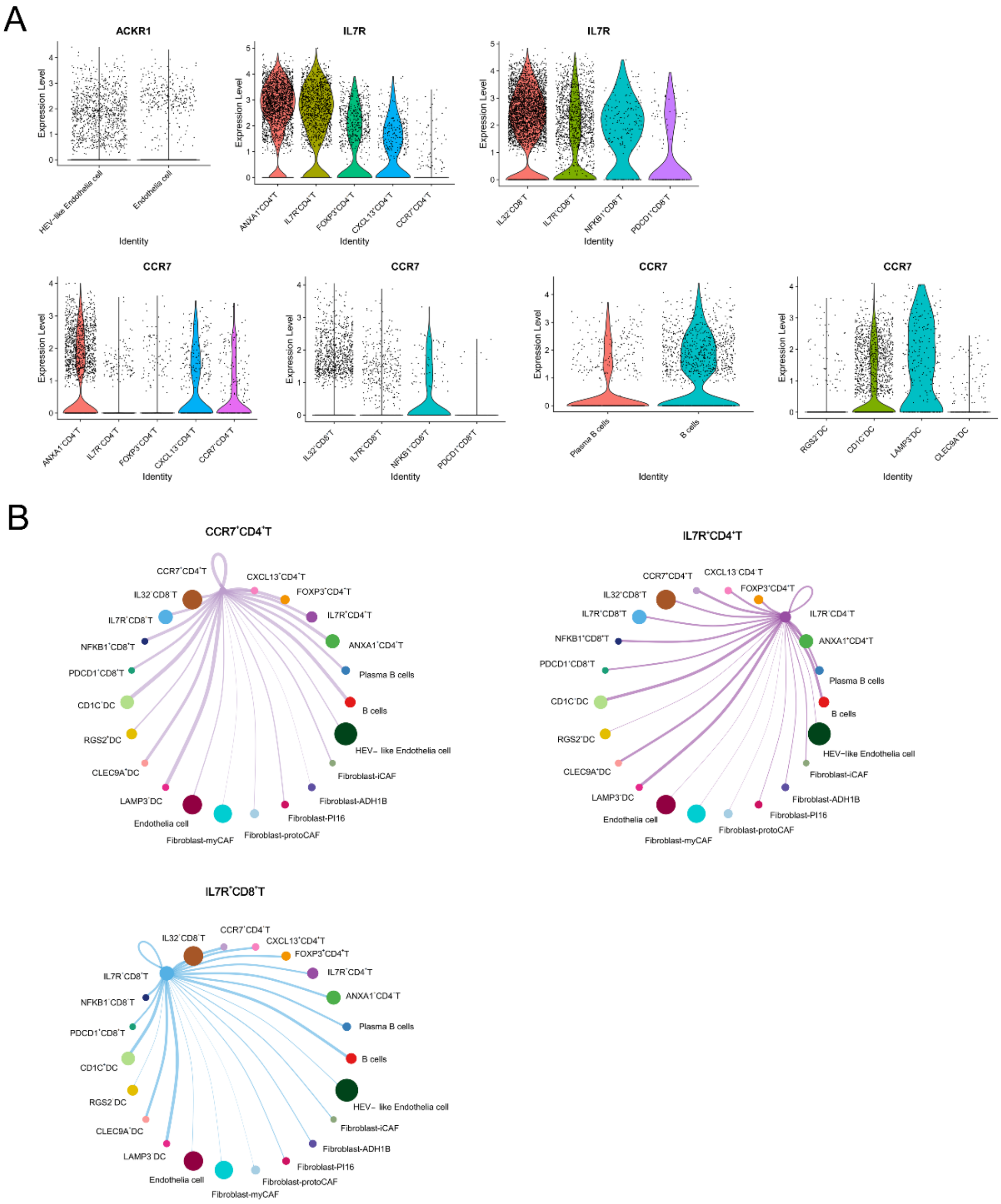

Supplementary Figure 13. (A) Representative HE-stained pathological section (HCC-2L) from the HRA000437 dataset; (B) Spatial mapping of endothelial cells, T cells, B cells, and DCs on the pathological section; (C) Spatial expression patterns of ACKR1, CCR7, and IL7R. (D-F) Corresponding analyses for HCC-4L. DCs, dendritic cells

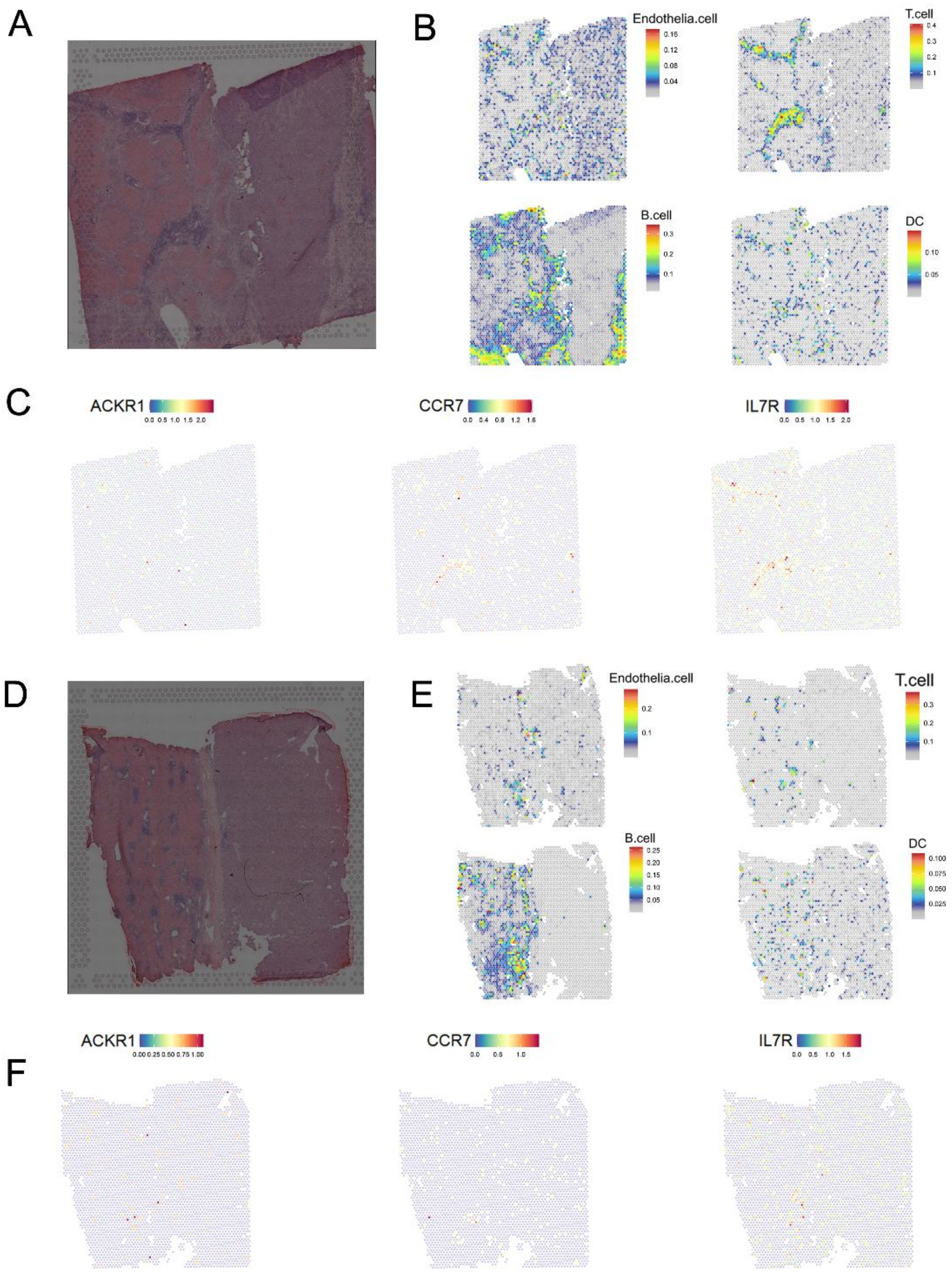

Supplementary Figure 14. Representative HE-stained pathology sections and spatial expression patterns of ACKR1, CCR7, and IL7R in responders (A) and non-responders (B).

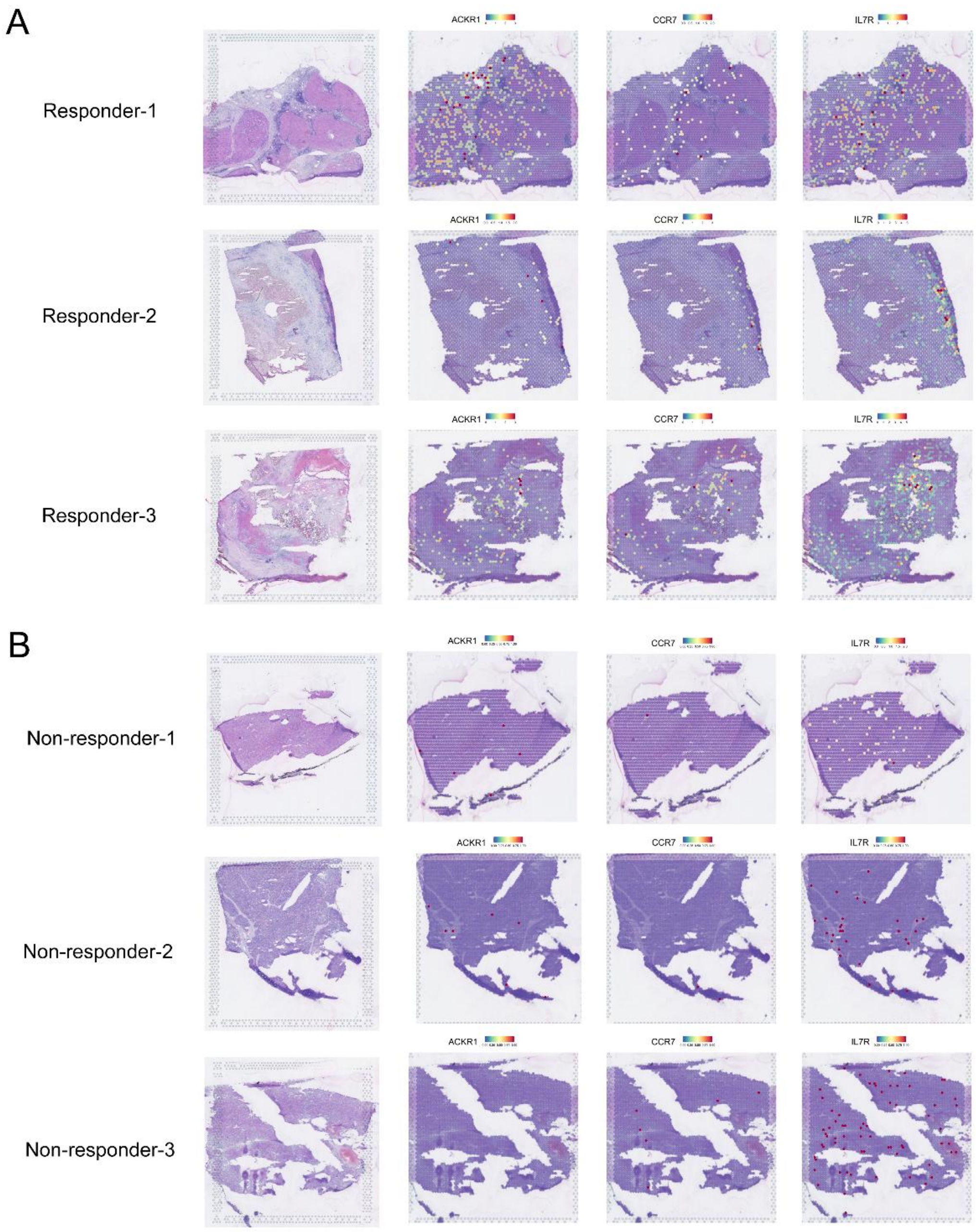

Supplementary Figure 15. (A) Representative mIHC images of TLS in the GXMUCH cohort; (B) Identification of characteristic TLS: Agg (lymphocyte aggregates), FL1 (primary lymphoid follicles), and FL2 (secondary lymphoid follicles). TLS, tertiary lymphoid structure; HE, hematoxylin-eosin; mIHC, multiplex immunohistochemical.

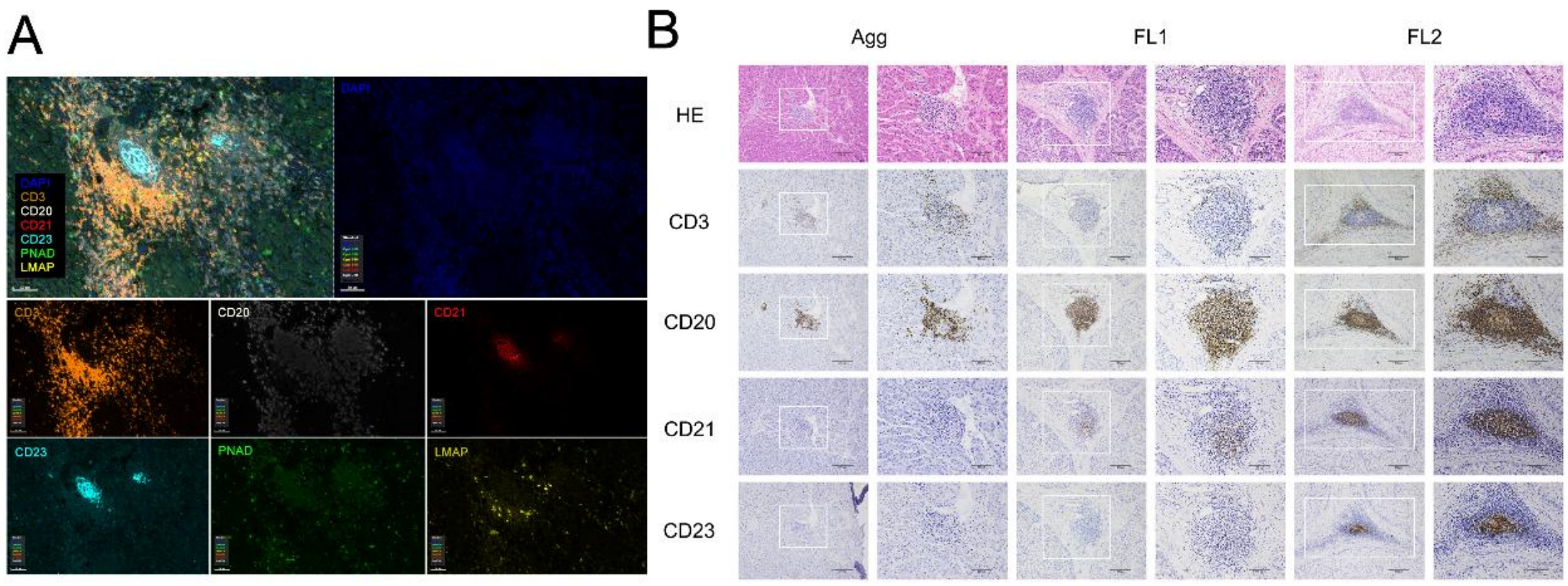

References

1. Finkin S, Yuan D, Stein I, et al. Ectopic lymphoid structures function as microniches for tumor progenitor cells in hepatocellular carcinoma. *Nat Immunol*. Dec 2015;16(12):1235-44.

2. Langfelder P, Horvath S. WGCNA: an R package for weighted correlation network analysis. *BMC Bioinformatics*. Dec 29 2008;9:559.

3. Tibshirani R. The lasso method for variable selection in the Cox model. *Stat Med*. Feb 28 1997;16(4):385-95.
